# Machine learning models for estimating the overall oil recovery of waterflooding operations in heterogenous reservoirs

**DOI:** 10.1038/s41598-025-97235-5

**Published:** 2025-04-26

**Authors:** Sayed Gomaa, Ahmed Ashraf Soliman, Mohamed Mansour, Fares Ashraf El Salamony, Khalaf G. Salem

**Affiliations:** 1 Mining and Petroleum Engineering Department, Faculty of Engineering, Al-Azhar University, Cairo, Egypt; 2 Petroleum Engineering and Gas Technology Department, Faculty of Energy and Environmental Engineering, British University in Egypt (BUE), El Sherouk City, Cairo, Egypt; 3 Artificial Intelligence Department, Faculty of Informatics and Computer Science, British University in Egypt (BUE), El Sherouk City, Cairo, Egypt; 4 Department of Reservoir Engineering, South Valley Egyptian Petroleum Holding Company (GANOPE), Cairo, Egypt

**Keywords:** Waterflooding, Oil recovery, Machine learning, Artificial neural network, Mobility ratio, Reservoir permeability variation, Water-oil ratio, Initial water saturation, Fossil fuels, Chemical engineering, Environmental sciences, Scientific data, Statistics

## Abstract

Waterflooding is the most widely used improved oil recovery technique. Predicting the overall oil recovery resulting from waterflooding in oil reservoirs is crucial for effective reservoir management and appropriate decision-making. Machine learning (ML) techniques present resourceful and fast-track tools, aiding in predicting oil recovery, which is time-consuming and costly to accomplish by simulation studies. In this paper, four machine learning models: artificial neural network (ANN), Random Forest (RF), K-Nearest Neighbor (K-NN), and Support Vector Machine (SVM) are applied to estimate the overall oil recovery (R) of water flooding. Initially, statistical methods were employed to analyze the input data before applying machine learning techniques. These models take into consideration the mobility ratio (M), reservoir permeability variation (V), water-oil production ratio (WOR), and initial water saturation (S_Wi_). 1054 datasets were utilized to develop machine-learning models. ANN-based correlation was developed to estimate the overall oil recovery of waterflooding. The ANN proposed model achieves a high coefficient of determination (R^2^) of 0.999 and a low root-mean-square error (RMSE) of 0.0063 on the validation dataset. On the other hand, the other machine learning models like RF, K-NN, and SVM achieve accurate estimation of overall oil recovery (R), where the coefficients of determination (R^2^) values are 0.97, 0.95, and 0.80 and the RMSE scores are 0.0282, 0.0405, and 0.0629 on the validation dataset, respectively. The innovative application of such ML models demonstrates significant improvements in prediction accuracy and reliability, offering a robust solution for optimizing oil recovery processes. These machine learning models provide the industry and research with efficient and economical tools for accurately estimating oil recovery in waterflooding operations within heterogeneous reservoirs.

## Introduction

### Background

Oil is typically produced from reservoirs through three recovery stages: primary, secondary, and tertiary^1^. Initially, the primary recovery normally involves the production of a reservoir assisted by the natural energy of the reservoir. Practically, the average recovery factor for the primary recovery is less than 30% of the initial oil in place^2^. Therefore, when natural energy is depleted (decline of reservoir pressure), additional energy must be applied to the reservoir to support the reservoir pressure and maintain oil production through secondary recovery. Such secondary recovery can be conducted by water or gas injection for pressure maintenance/support. Waterflooding is the most widely used method for secondary oil recovery to enhance oil production and level up the reservoir pressure^3^. Practically, waterflooding is still one of the most potential recovery methods, recovering around one-third of the original oil in place in conventional oil fields^4^. Lastly, the tertiary recovery stage or enhanced oil recovery (EOR) is applied to increase the oil recovered beyond the range that primary and secondary recovery can reach^5,6^. Typically, EOR methods may be categorized into four classes: chemical^7–10^, thermal^11^, miscible gas^12^, and others^13,14^.

In the waterflooding process, water is injected into reservoirs, and the displaced oil is produced from nearby production wells. The efficiency of water to displace oil from permeable and porous reservoirs is known as overall oil recovery. The overall oil recovery of waterflooding is mainly affected by the mobility ratio as well as the geology of the reservoir^15^. The ideal displacement, also known as piston-like displacement, is the most desirable characteristic, in which the total amount of conventional crude oil recoverable from a reservoir is obtained by injecting the same volume of water^16^. While non-ideal displacement is, unfortunately, more prevalent in nature, it is caused by the difference in viscosity between water (displacing phase) and oil (displaced phase). In this case, the oil will be bypassed as the water pushes it through the reservoir since the water can move faster than the oil. The development of water fingering or coning results in an unfavorable displacement and may reduce the overall oil recovery^17^.

Various factors influence the process of displacing oil with water. These include the relative permeability of oil and water, the viscosities of the fluids, the heterogeneity of the reservoir, the distribution of pore sizes, capillary pressure, fluid saturations, and the distance between the injection and production wells^18–20^. All of these factors affect the overall oil recovery. The total oil recovery (R) for any secondary or tertiary recovery method is determined by the product of three distinct efficiency factors, as illustrated below^21^:

1 $$\:\mathbf{R}={\mathbf{E}}_{\mathbf{D}}{\mathbf{E}}_{\mathbf{A}}{\mathbf{E}}_{\mathbf{V}}$$

Displacement sweep efficiency (ED) refers to the ratio of the volume of oil displaced by water from small pores in a reservoir to the volume of oil originally present in those small pores before the displacement process^22^. E_D_ is affected by pore geometry, fluid distribution, wettability of the rock, and the saturation of the reservoir. Besides, the displacement efficiency is also affected by the viscosity of oil, which decreases with increasing oil viscosity^23^. Welge (1952)^24^ presented an analytical model for computing the displacement efficiency at water breakthrough by drawing a tangent line to the fractional flow curve starting from connate water saturation (F_w_ versus S_w_). In addition, various authors have applied the fractional flow theory to waterflooding^25^, polymer flooding^26^, nanofluid flooding^27^, and alkaline-surfactant-polymer^28^.Areal sweep efficiency (EA) is defined as the proportion of the area swept by water to the total area^21^. EA is influenced by two primary factors: the well pattern and the mobility ratio of the fluids in the reservoir. Consequently, a lower mobility ratio leads to higher areal sweep efficiency^29^.Vertical sweep efficiency (EV) denotes the proportion of the vertical sections of the pay zone that are reached by the displacing fluid. The main factors influencing vertical sweep efficiency are fluid mobilities, the extent of gravity segregation, the water-oil production ratio, the vertical heterogeneity of the reservoir, and the total volume of injected fluid^30^.

### Literature review

Prediction of water flooding performance was presented in the literature using analytical solutions. Stiles (1949)^31^ introduced the first model to estimate waterflooding performance in stratified oil reservoirs. The key assumption of this model is that the velocities in different layers are proportional to their absolute permeabilities, with water breaking through first in the most permeable layers. Additionally, it assumes a piston-like displacement pattern between the displacing fluid (water) and the displaced fluid (oil). Dykstra and Parsons (1950) created an empirical model to evaluate the efficiency of waterflooding in stratified oil reservoirs with non-communicating layers^32^. This model assumes immiscible and piston-like displacement, disregarding the effects of gravity. It uses the water-oil production ratio (WOR), reservoir permeability variation (V), and water-oil mobility ratio (M) as correlation parameters. They also introduced vertical coverage correlation charts for log-normal permeability distributions based on the mobility ratio and the coefficient of reservoir permeability variation at different water-oil production ratios. Building on their work, Johnson (1956) developed a set of correlation charts for overall oil recovery at various water-oil production ratios^33^.

Yokoyama et al. (1981)^34^ investigated how capillary pressure affects waterflooding performance. They used a two-layer simulation model with water injected at one end and an oil-water mixture produced at the other, due to the complexity of the issue. In a homogeneous medium, longitudinal capillary pressure reduces waterflood oil recovery, a condition unlikely to occur in field-scale floods. Conversely, transverse capillary pressure increases waterflood oil recovery in stratified media, a condition likely to be achieved in field-scale floods. El-Khatib (1985)^35^ introduced a mathematical correlation to estimate waterflooding performance for both communicating and non-communicating layers. The study examined the effects of crossflow, mobility ratio, porosity, fluid saturation, and permeability distribution on waterflooding performance. The model predicts water cut, oil recovery, total injected fluid volume, and changes in the injection rate at breakthrough. El-Khatib (1999)^36^ developed an analytical solution to calculate waterflooding performance in communicating stratified systems with log-normal permeability distributions. By integrating various variables, he created a single chart that encompasses all water-oil production ratios, permeability variations, and water-oil mobility ratios, eliminating the need for separate charts for each ratio. Furthermore, El-Khatib (2001)^37^ used the Buckley-Leverett frontal advance theory to formulate a mathematical model for estimating waterflooding performance in non-communicating stratified reservoirs. He presented an effective water-oil mobility ratio based on the average total mobility in the invaded zone to account for variable saturation behind the displacement front. El-Khatib (2003)^38^ developed a mathematical model to predict waterflooding performance in communicating stratified reservoirs, considering the vertical gravitational crossflow due to oil-water density differences. The findings showed that gravity crossflow delays water breakthrough in highly permeable layers, enhancing oil recovery and reducing water cuts. Following the Dykstra-Parsons approach, it is assumed that the reservoir layers are horizontal. El-Khatib (2012)^39^ established a correlation waterflooding performance in inclined reservoirs by modifying the Dykstra-Parsons equation. This modification introduced a dimensionless gravity number that accounts for the dip angle effect and the density differences between displacing fluids like water and displaced fluids like oil.

Craig et al. (1971)^23^ developed a graphical model to estimate the areal sweep efficiency at water breakthrough based on the mobility ratio for a five-spot flooding pattern. Willhite (1986)^40^ converted Craig’s graphical correlation into a mathematical model, which is as follows:

2 $$\:{\varvec{E}}_{\varvec{A}\varvec{B}\varvec{T}}=0.54602036+\frac{0.03170817}{\varvec{M}}+\frac{0.30222997}{{\varvec{e}}^{\varvec{M}}}-0.00509693\varvec{M}$$

After the water breakthrough, the areal sweep efficiency improves due to the expansion of the total swept area as water injection continues. Dyes et al. (1954)^19^ developed the following relationship between the increase in the efficiency of areal sweep and the ratio of water volume injected at any time after the water breakthrough:

3 $$\:{\varvec{E}}_{\varvec{A}}={\varvec{E}}_{\varvec{A}\varvec{B}\varvec{T}}+0.2749\mathbf{l}\mathbf{n}\left(\frac{{\varvec{W}}_{\varvec{i}}}{{\varvec{W}}_{\varvec{i}\varvec{B}\varvec{T}}}\right)$$

Furthermore, Dyes et al. (1954) provided graphical correlations that relate the areal sweep efficiency at and after water breakthrough with the reciprocal mobility ratio and the water cut for various injection well patterns, including five-spot, direct line, and staggered line patterns.

Fassihi (1986)^41^ extracted the data from the graphical correlations of Dyes et al. (1954) and established a mathematical model to calculate the efficiency of the areal sweep as a function of water cut and mobility ratio for five-spot, direct line, and staggered line patterns (Eq. (4)). The correlation coefficients from Fassihi (1986) are shown in Table 1.

4 $$\:{\varvec{E}}_{\varvec{A}}=\frac{1}{1+[\varvec{a}\mathbf{l}\mathbf{n}(\varvec{M}+\mathbf{b})+\mathbf{c}]{\varvec{f}}_{\varvec{w}}+\mathbf{d}\mathbf{l}\mathbf{n}(\varvec{M}+\mathbf{e})+\mathbf{f}}$$

**Table 1:** The correlation coefficients of Fassihi (1986)^41^.

Coefficients	Direct line-drive	Staggered line-drive	Five-spot
a	−0.3014	−0.2077	−0.2062
b	−0.1568	−0.1059	−0.0712
c	−0.9402	−0.3526	−0.511
d	0.3714	0.2608	0.3048
e	−0.0865	0.2444	0.123
f	0.8805	0.3158	0.4394

Recently, machine learning techniques were used to predict the performance of water flooding. In this regard, Kalam et al. (2021)^42^ presented a new empirical model based on ANN to estimate the five-spot waterflood performance in a heterogenous reservoir at and after water breakthrough. The developed ANN model can predict the overall oil recovery accurately in terms of the wettability of rock, mobility ratio, permeability variation coefficient, anisotropy ratio, and production water cut. Moreover, the ANN model could be a suitable tool for 5-spot waterflooding heterogeneous reservoirs and waterflooding assessment before building a reservoir simulation model.

Gomaa et al. (2022)^43^ developed new accurate correlations to predict the vertical sweep efficiency based on the water-oil production ratio, reservoir permeability variation, and mobility ratio using Nonlinear Multiple Regression (NLMR) and Artificial Neural Networks (ANN). The neural network model achieved a high coefficient of determination (R²) of 0.999 and a low mean square error of 0.0001.

In another study, Gomaa et al. (2022)^44^ established ANN models to calculate the efficiency of the areal sweep at and after water breakthrough for different injection well patterns such as five-spot, nine-spot, direct line, and staggered line patterns. The ANN models for calculating the efficiency of areal sweep for five-spot, direct line, and staggered line patterns are developed as a function of mobility ratio and water cut, while the ANN model for estimating the E_A_ of the regular nine-spot pattern is developed as a function of mobility ratio, producing ratio, and water cut.

### The gap and work objective

In contrast to much of the existing literature on analytical solutions and ML models, this study predicts the overall oil recovery of waterflooding using four distinct machine-learning techniques. Moreover, the previous studies were mostly focused on applications of artificial neural networks in the prediction of oil recovery^42–44^. To the author’s best knowledge, the developed machine learning techniques and the gathered database for prediction of overall oil recovery of waterflooding are so far the most comprehensive of its kind. These machine learning models provide the industry and researchers with an efficient and economical means to accurately estimate the overall oil recovery of waterflooding operations in heterogeneous reservoirs. The primary objective of this research is to utilize four distinct machine learning techniques: Artificial Neural Networks (ANN), Random Forests (RF), K-Nearest Neighbors (K-NN), and Support Vector Machines (SVM) to estimate the overall oil recovery from waterflood operations in heterogeneous reservoirs. This study introduces innovative and more efficient methods for predicting the overall oil recovery from waterflooding, surpassing traditional analytical based on 1054 datasets of mobility ratio, reservoir permeability variation, water-oil production ratio, and initial water saturation. The machine learning (ML) models developed in this research can be readily applied to new datasets, enabling accurate predictions of oil recovery. These established ML models also provide a robust foundation for further enhancements. As more data becomes available, the models’ accuracy and reliability are expected to improve. Machine learning techniques have been extensively utilized to predict the recovery performance in several recovery processes, such as waterflooding in heavy oil reservoirs^45^, low-salinity and hybrid low-salinity chemical flooding^46,47^, flooding in stratified reservoirs^48^, CO_2_ flooding in sandstone reservoirs^49^, immiscible flooding in heterogeneous reservoirs^50^, polymer and surfactant-polymer flooding^51,52^, and steam-assisted gravity drainage (SAGD)^53^.

## Methodology

**Fig. 1:**
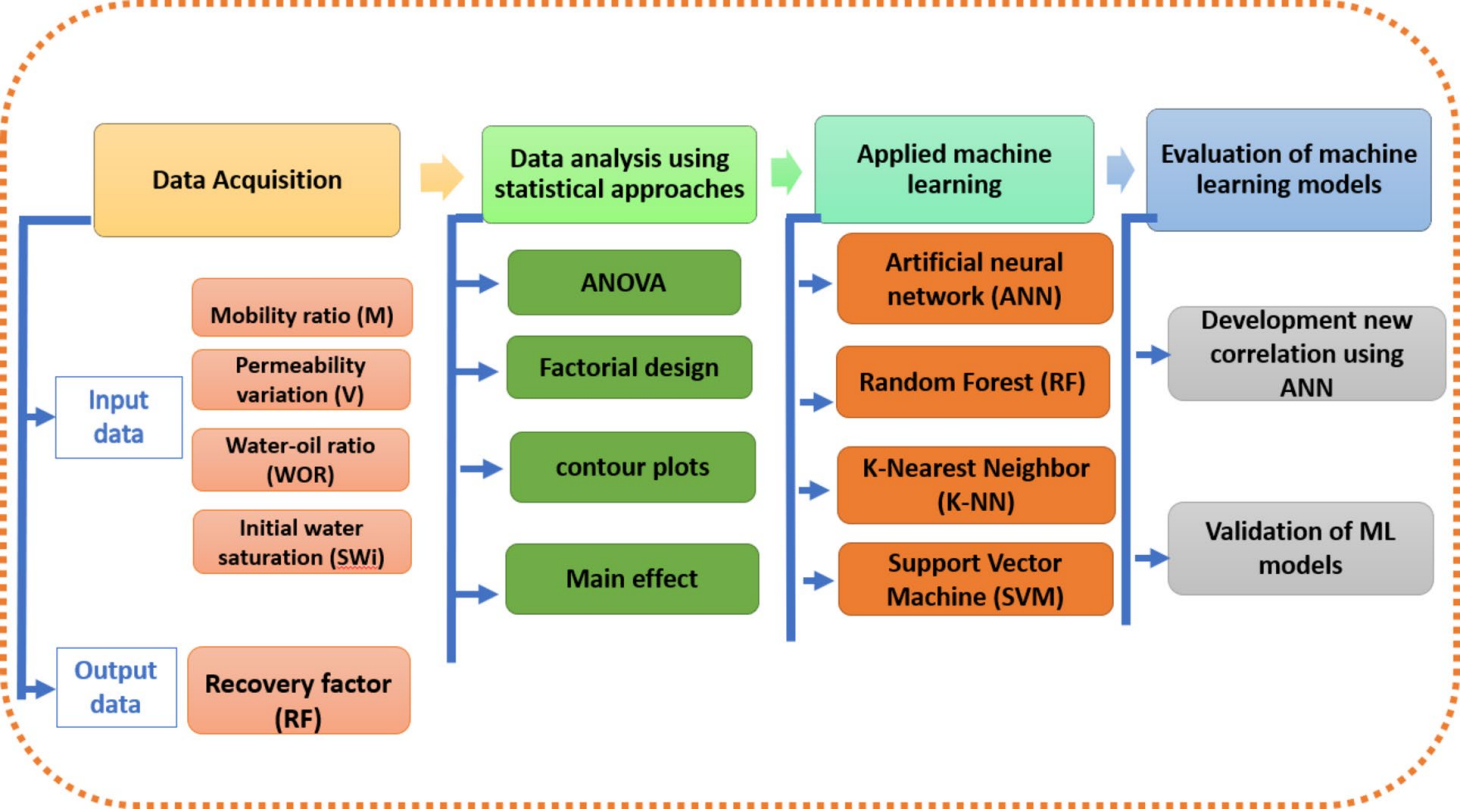
Research methodology for prediction of oil recovery of waterflooding using ML.

The research’s procedural approach is schematically shown in Fig. 1 and explained in the following points.

### Data acquisition

**Fig. 2:**
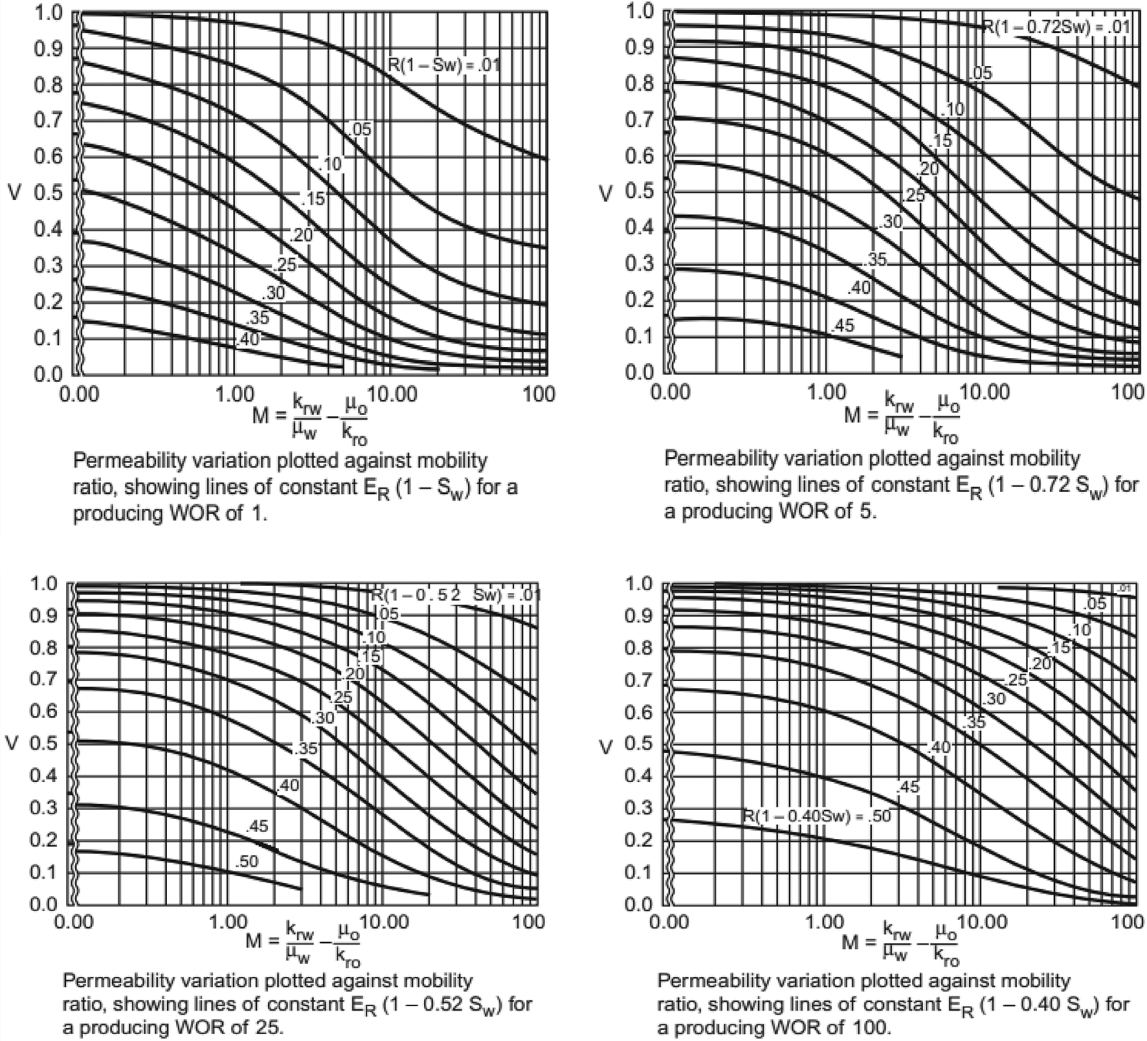
Simplified Dykstra and Parsons curves. Reprinted with permission from Johnson Jr, C. E^33^. Copyright (1949) Society of Petroleum Engineers.

1054 datasets are extracted from literature specifically Johnson correlations^33^ (Fig. 2**)** to build machine learning models for estimating the overall oil recovery (R) in terms of mobility ratio (M), reservoir permeability variation (V), water-oil production ratio (WOR), and initial water saturation (S_Wi_). These parameters were chosen because they collectively capture the essential physical and fluid dynamics that govern the water flooding process, thereby enabling the model to make accurate predictions of oil recovery. We adopted stringent criteria to ensure the quality of the data collected. The first step involved gathering relevant data and including essential features that influence the recovery factor of water flooding. Ensuring data quality was paramount, which involved considering factors such as accuracy, consistency, and distribution. Additionally, we collected a substantial volume of data (1041 datasets) to train a robust and reliable model. Finally, we included diverse datasets to capture a wide range of reservoir conditions, thereby enhancing the model’s generalizability and effectiveness in various scenarios.

**Fig. 3:**
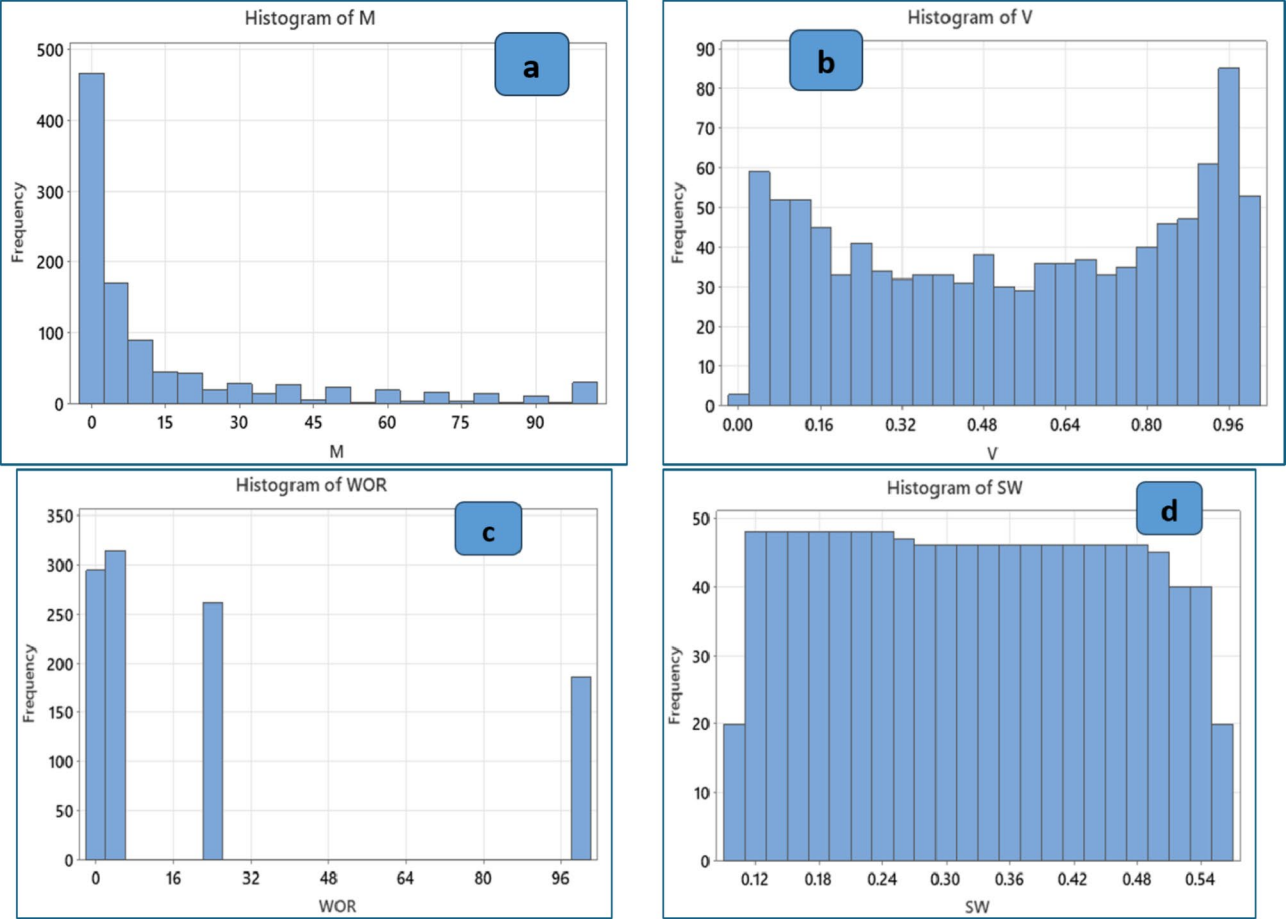
Histogram plots of input variables based on the gathered data from the literature: (**a**) mobility ratio (M), (**b**) reservoir permeability variation (V), (**c**) water-oil production ratio (WOR), and (**d**) initial water saturation (SWi).

**Fig. 4:**
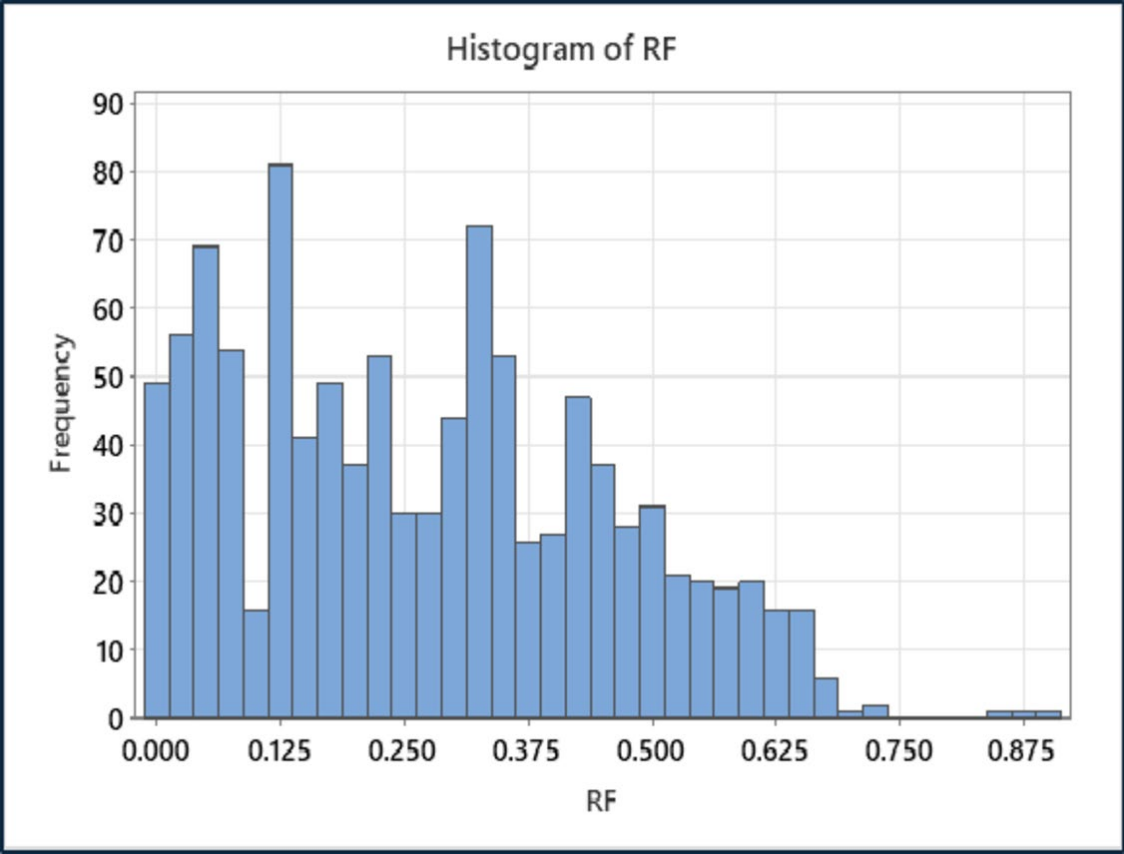
Histogram plots of output variable based on the gathered data from Jhonson correlation: Recovery factor.

As indicated in Table 2, the statistical analysis involves specifying the mean, median, mode, minimum, and maximum values and dispersion parameters such as standard error, standard deviation, kurtosis, and skewness. According to Table 2, the mobility ratio ranges from 0.098 to 100, the reservoir permeability variation ranges from 0.006 to 0.998, the water-oil production ratio ranges from 1 to 100, the initial water saturation ranges from 0.1 to 0.55, and the overall oil recovery ranges from 0.011 to 0.889. The dataset was graphically displayed, and the sampling distribution was explained using histograms. Figure 3 (a-d) shows the histograms of the data sets for mobility ratio (M), reservoir permeability variation (V), water-oil production ratio (WOR), and initial water saturation (SWi), respectively. The datasets encompass a variety of mobility ratios from 0.09 up to 100 (unfavorable M). However, most of the data points range from 1 to 15. Furthermore, the data on reservoir permeability variation (V) showed a large range of values (0.006–0.998) that is mean from very homogeneity to very heterogeneity. Regarding the water-oil production ratio (WOR), the dataset comprises frequent values of 1, 5, 25, and 100. Furthermore, the initial water saturation (SW_i_) data had an extensive range of values (0.1 to 0.55). Figure 4 depicts the histogram of the overall oil recovery from water flooding. A variety of oil recovery are covered by the datasets, but most of the data points lie between 10% and 50% psi. Based on the datasets, the average oil recovery after water flooding (WF) was 27%.

**Table 2:** Statistical analysis of the datasets.

	Parameters				
	M	V	WOR	S_Wi_	R
Mean	16.431	0.538	25.602	0.32	0.271
Standard error	0.797	0.01	1.099	0.004	0.006
Median	3.64	0.556	5	0.32	0.25
Mode	100	0.988	5	0.11	0.313
SD	25.881	0.319	35.678	0.131	0.185
SV	669.818	0.101	1272.946	0.017	0.034
Kurtosis	2.695	-1.4	0.447	-1.193	-0.713
Skewness	1.915	-0.099	1.456	0.036	0.395
Range	100.214	0.992	99	0.45	0.878
Minimum	0.098	0.006	1	0.1	0.011
Maximum	100	0.998	100	0.55	0.889
Count	1054	1054	1054	1054	1054

**Fig. 5:**
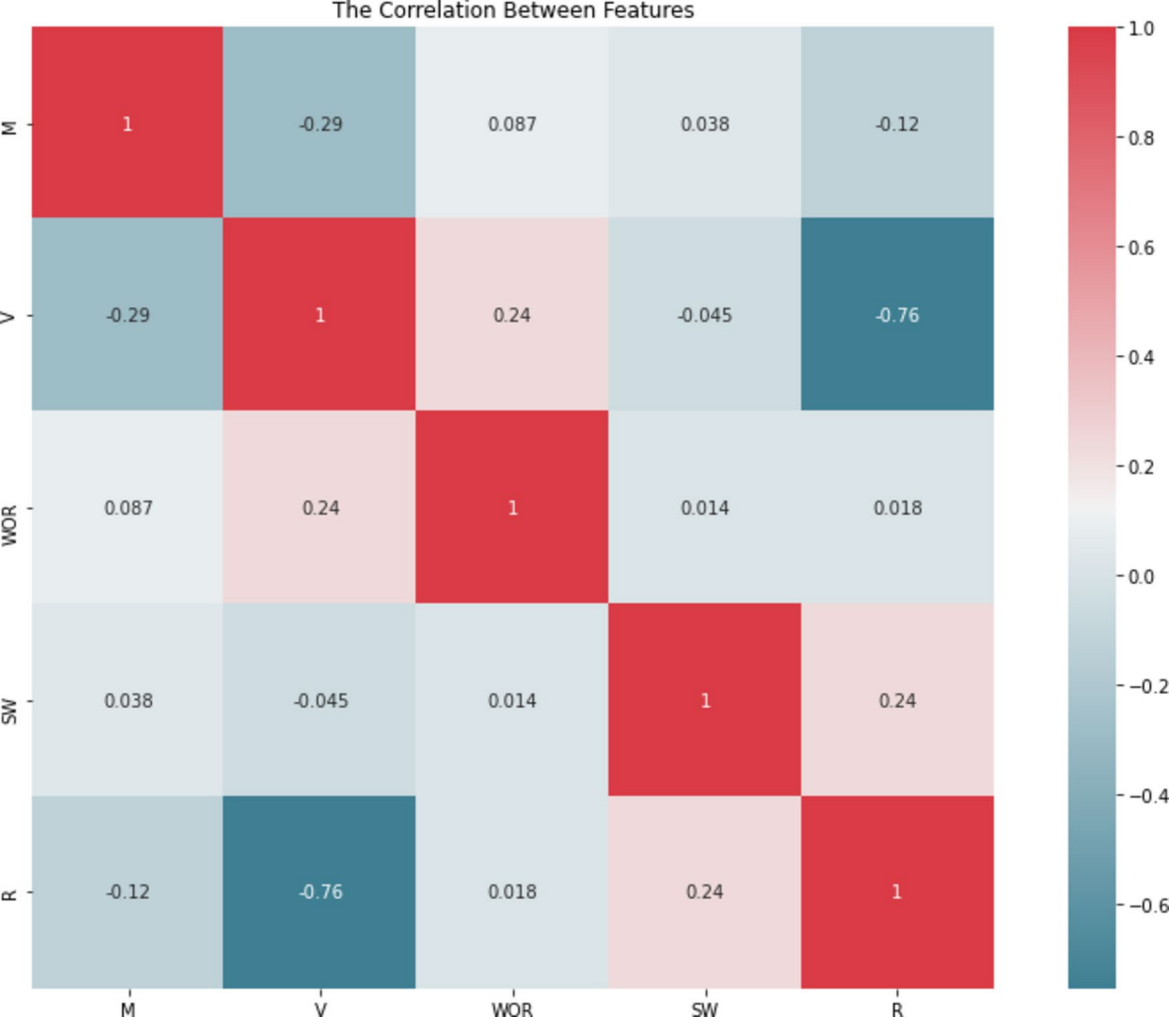
Relative importance of input parameters with overall oil recovery (R).

As shown in Fig. 5, the correlation map shows the relation between the features with each other and with the target value (R). The S_Wi_ feature has a significant positive relationship with the target value (R) while the feature V has a significant negative relationship with the target value (R), which is thought to be the most impactful feature in the dataset.

### Data analysis using statistical approaches

Before ML models were developed, the gathered datasets were examined through statistical tools to authenticate the relations between the input parameters and output (oil recovery). A predictive machine learning (ML) model’s accuracy heavily depends on the quality of the input dataset^54^. It’s important to note that the prediction accuracy of ML models is validated by examining the significance of input features on the output data. Consequently, an analysis of variance (ANOVA) test was conducted using Minitab software to assess several variables of the generated model. The relationship between oil recovery from water flooding and the input variables was analyzed using P and F values. A p-value of less than 0.05 indicates the statistical significance of a factor. Furthermore, factorial design, contour plots, and main effects plots were created and discussed to emphasize the impact of input data on oil recovery from water flooding^54^.

### Applied machine learning models

Numerous machine learning models are designed for regression tasks, including artificial neural networks, random forests, K-nearest neighbors, and support vector machines. All of these models can be implemented in a regression task, but each model performs differently from the other models due to the completely different mechanisms of each machine learning model. Each machine learning model possesses a unique set of parameters that can be adjusted to produce different iterations of the same model, and each iteration will perform differently, resulting in varied scores.

One widely used machine learning method is the artificial neural network (ANN), which mimics the way the human brain learns. An ANN consists of three layers: an input layer, one or more hidden layers, and an output layer. Each layer comprises numerous interconnected nodes (neurons) that connect the input layer to the output layer through the hidden layer(s). The output for each neuron is determined by a nonlinear function of the sum of its inputs^55^. The neural network’s edges and nodes contain weights that are adjusted during the learning phase. ANNs have been applied in various petroleum engineering contexts, such as reservoir engineering, where they are used to estimate water saturation and permeability in limestone and dolomite formations^56,57^ and estimate the dewpoint pressure for retrograde gas condensate reservoirs^58^. Besides, Applications of ANN In drilling engineering include estimating the pressures (pore and fracture pressures) while drilling for mixed lithologies^59^ and predicting the yield point and plastic viscosity of the invert-emulsion mud^60^.

The K-Nearest Neighbour (K-NN) machine learning method is extensively employed for classification and regression tasks. This model aims to measure the distance between a new, unlabelled data point and existing training data points in the feature space, which is essential for class prediction. During this phase, the nearest data points are arranged based on the k-value of the new observation. The k-value, a hyperparameter in this context, is used to identify the k-nearest data points for the new observation. The K-NN classifier then votes and assigns the predicted class to the new unlabelled data sample based on the number of class labels among the k-neighbours^61^.

The support vector machine (SVM), also referred to as the kernelized support vector machine (KSVM), was introduced by Cortes and Vapnik^62^. SVMs have supervised machine learning algorithms that analyze a dataset’s many inputs to create a decision boundary (or hyperplane) between many classes. As a consequence, a single or multiple feature vector may be used to predict labels. Because of its arrangement, data points close to each class are kept at a safe distance. The term “support vector machine” pertains to the data points that are nearest, which are referred to as support vectors. The fundamental purpose of this approach is binary linear classification and prediction. This method has been employed effectively in several biological applications. SVMs are widely utilized in biomedical practice to characterize microarray gene expression patterns^62^.

A random forest (RF) is an ensemble model for classification and regression that includes several models and is compatible with a wide range of datasets. These models include Bootstrap aggregation and bagging. To avoid overfitting, Bagging can minimize the variance of the model and improve the generalization. Even without the use of decision trees, this approach can resolve significant conflicts. In a random forest model, multiple decision trees (DTs) are used, each with slight variations from one another. For each data point, the multiple results from the decision trees are combined. During the integration process, a majority vote is used for classification tasks, while an average value is calculated for regression tasks. In terms of predictions, combined decision trees outperform single decision trees since they are all trained individually on random samples from a training dataset. Because it employs randomness in tree building to ensure that all trees are unique from one another, this model is termed a random forest^63^.

### Data splitting

Before the data were fitted into the models, some pre-processing steps took place. For building an ANN, K-NN, SVM, and random forest models to estimate the overall oil recovery. The dataset was divided into training and validation subsets, with 70% allocated for training and the remaining 30% for validation. Additionally, the dataset was standardized using the StandardScaler technique to achieve a uniform value ratio, which helps the models avoid confusion due to large variances in data values. Other scaling methods like Robust Scaler, which uses the median and the interquartile range, could also be considered for data with many outliers. In the current study, the data had minimal outliers, making Standard Scaler a suitable choice. The StandardScaler is a popular preprocessing tool in machine learning used to standardize features by removing the mean and scaling to unit variance. This ensures that each feature contributes equally to the model, which can improve the performance and convergence speed of many machine learning algorithms. StandardScaler transforms the data such that the distribution of each feature has a mean of 0 and a standard deviation of 1. This is particularly useful when the features have different units or scales. The transformation is given by the following equation:

5 $$\:Z=\frac{x-\mu\:}{\sigma\:}\:\:\:\:\:\:\:\:\:\:\:\:\:\:\:\:\:\:\:\:\:\:\:\:\:\:\:\:\:\:\:\:\:\:\:\:\:\:\:\:\:\:\:\:\:\:\:\:\:\:\:\:\:\:\:\:\:\:\:\:\:\:\:\:\:\:\:\:\:\:\:\:\:\:$$

Where z is the standardized value, x is the original value, µ is the mean of the feature, σ is the standard deviation of the feature.

### Models evaluation and error analysis

Various traditional statistical measures and graphical error analyses are employed to evaluate the precision of the model. These methods help in assessing the accuracy, validity, and reliability of the developed models, as well as in predicting the performance of the machine learning algorithms created^54^. Furthermore, relative error distribution graphs and Cross-plots were also employed. In the cross plots, the predicted and experimental data points are plotted against each other to evaluate the model’s accuracy in predicting the experimental results. The effectiveness of the diagram is assessed by examining how closely the data points cluster around the equality line and their deviation from the 45° line.

#### Statistical error analysis

In this study, the reliability of the developed paradigms was evaluated using several statistical measures, including ARE%, AARE%, RMSE, SD, and R²^64,65^. The following formulas are used to compute these statistical parameters. The following formulas are used to compute these statistical parameters.

6 $$\:ARE=\frac{100}{N}\:\:\sum\:_{i=1}^{N}\frac{{y}_{i\:actual}-{y}_{i\:predicted}}{{y}_{i\:actual}}\:\:\:\:\:\:\:\:\:\:\:\:\:\:\:\:\:\:\:\:\:\:\:\:\:\:\:\:\:\:\:\:\:\:\:\:\:\:\:\:\:\:\:\:\:\:$$

7 $$\:AARE=\frac{100}{N}\:\:\sum\:_{i=1}^{N}\left|\:\frac{{y}_{i\:actual}-{y}_{i\:predicted}}{{y}_{i\:actual}}\right|\:\:\:\:\:\:\:\:\:\:\:\:\:\:\:\:\:\:\:\:\:\:\:\:\:\:\:\:\:\:\:\:\:\:\:\:\:\:\:$$

8 $$\:MSE=\frac{1}{N}\:\:\sum\:_{i=1}^{N}{(y}_{i\:actual}-{y}_{i\:predicted\:})^2\:\:\:\:\:\:\:\:\:\:\:\:\:\:\:\:\:\:\:\:\:\:\:\:\:\:\:\:\:\:\:\:\:\:\:\:\:\:\:\:\:\:\:\:\:$$

9 $$\:RMSE=\sqrt{MSE}$$

10 $$\:{R}^{2}=1-\frac{\sum\:_{i=1}^{N}{(y}_{i\:actual}-{y}_{i\:predicted\:})^2}{\sum\:_{i=1}^{N}{(y}_{i\:average\:actual}-{y}_{i\:predicted\:})^2}$$

where N symbolizes several datasets; y _i Actual_, y _i Predicted_, and y _i average Actual_ symbolizes actual RF, predicted RF, and an average of actual values of RF, respectively.

## Results and discussion

### Analysis of the impact of key factors on the recovery factor using statistical approaches

#### The analysis of variance (ANOVA)

ANOVA is a statistical tool widely employed to analyze various issues in the upstream oil industry^54,66^. In this study, ANOVA was utilized to obtain a quantitative interpretation of the investigated parameters. The results, displayed in Table 3, indicate that all input data significantly impact oil recovery through water flooding, with P-values less than 0.05. Reservoir permeability variation (V) shows the highest F-value (4055.99), signifying its stronger influence compared to other parameters. Additionally, the mobility ratio, water-oil production ratio, and initial water saturation had substantial effects on oil recovery, with F-values of 870.44, 377.7, and 237.2, respectively.

**Table 3:** ANOVA for oil recovery by water flooding.

Source	DF	Adj SS	Adj MS	F-value	*P*-value
Model	4	29.192	7.2981	1116.78	0
Linear	4	29.192	7.2981	1116.78	0
M	1	5.688	5.6883	870.44	0
V	1	26.506	26.5057	4055.99	0
WOR	1	2.468	2.4683	377.7	0
SW	1	1.55	1.5501	237.2	0

#### Factorial design

**Fig. 6:**
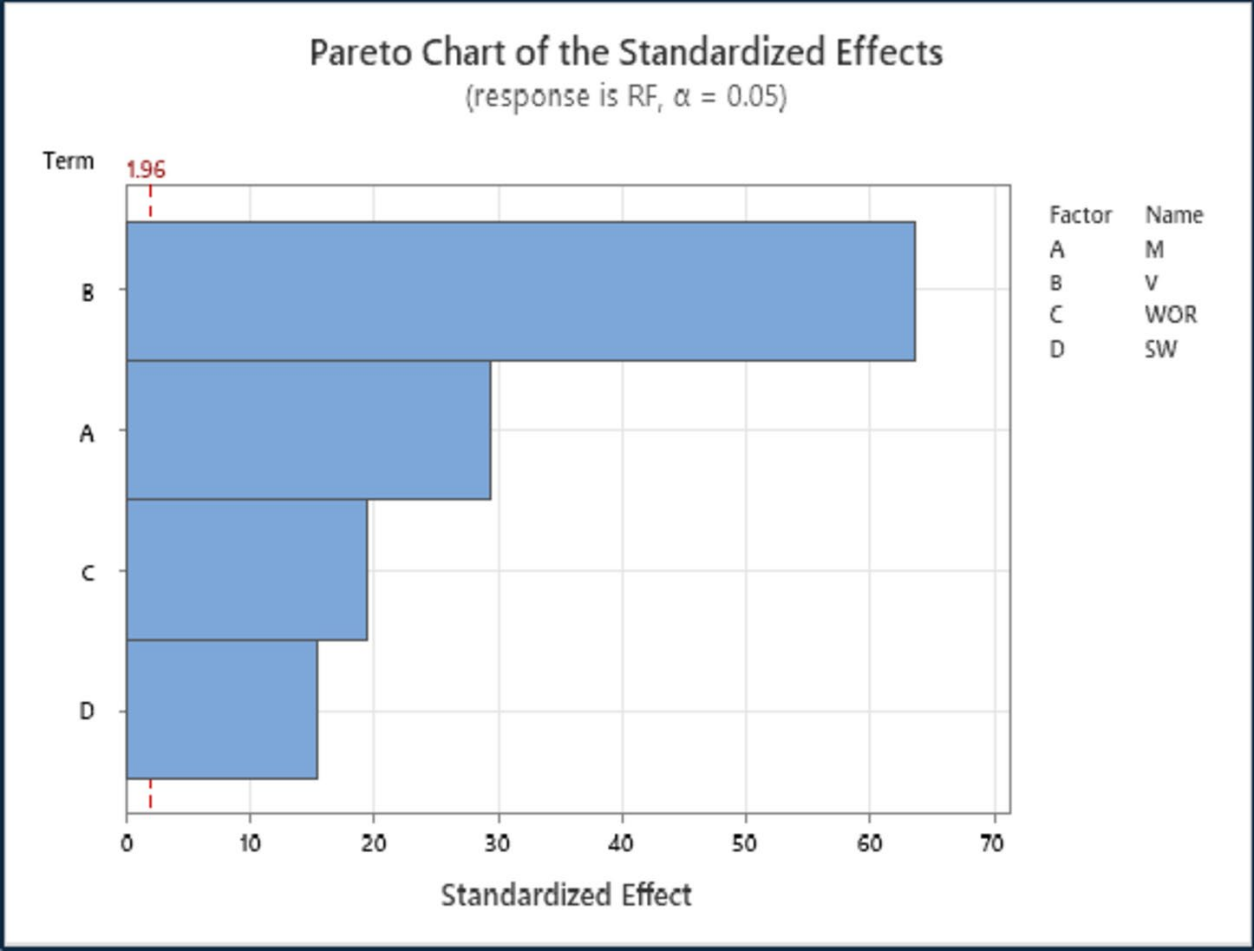
Pareto chart for results of oil recovery by water flooding.

Factorial design was utilized to identify the most significant factors among the study parameters. Minitab software was employed to conduct these statistical assessments. Generally, factorial design is a crucial statistical tool for examining the influence of various controllable elements on the response of interest. The results of the factorial design are presented in the Pareto diagram, which displays the effects of the factors from the highest to the lowest impact using horizontal bars. Additionally, a reference line on the Pareto chart indicates which effects are statistically significant. In this study, the analysis was performed for mobility ratio (M), reservoir permeability variation (V), water-oil production ratio (WOR), and initial water saturation (SWi). Figure 6 shows the Pareto chart for the water flooding recovery factor results. The overall effectiveness of these parameters, ranked from highest to lowest, was reservoir permeability variation (V) > mobility ratio (M) > water-oil production ratio (WOR) > initial water saturation (SWi). Furthermore, permeability variation (V) and mobility ratio (M) were the most dominant parameters affecting the recovery factor of water flooding. The statistical analysis using the Pareto chart is consistent with the previous analysis.

#### Contour plots

**Fig. 7:**
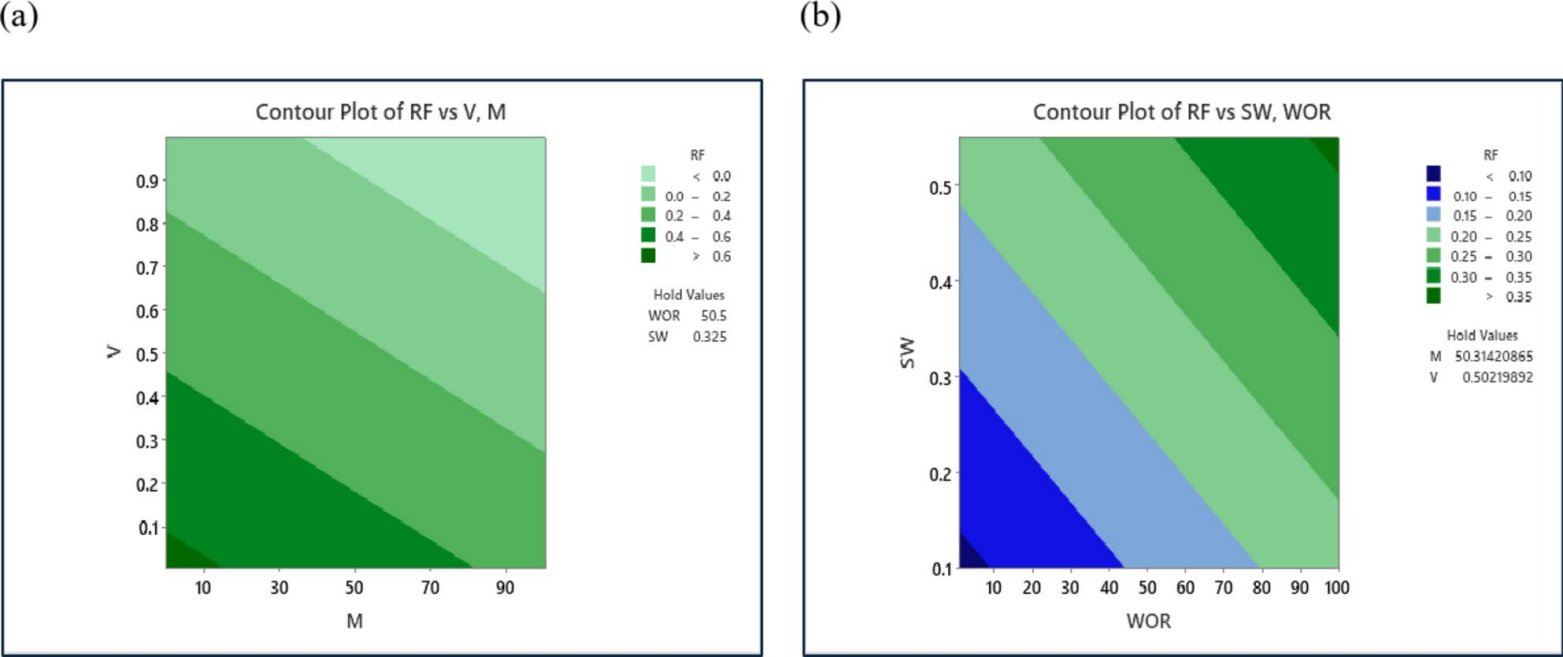
Contour map of recovery factor: (**a**) correlated with V and M, (**b**) correlated with Sw and WOR.

A contour plot was utilized to assess the impact of input variables on oil recovery. Figure 7a illustrates the contour plot for oil recovery via water flooding, analyzing the combined effects of the mobility ratio (M) and reservoir permeability variation (V). The plot indicates that the highest oil recovery values occur in regions with low mobility ratio and low permeability variation. Conversely, the lowest oil recovery values are observed in areas with high mobility ratio and high permeability variation. This figure is consistent and supportive from reservoir point of view. Figure 7b shows the contour plot for oil recovery by water flooding to analyze the mutual effects of water-oil production ratio, and initial water saturation. The valley in the lower left section of the graph indicates the lowest oil recovery values (< 0.1), corresponding to lower water-oil production ratios and initial water saturation. As the water-oil production ratio increases, oil recovery shifts to higher regions. Consequently, the upper right section of the graph signifies the highest oil recovery values, which align with the highest water-oil production ratios.

#### Main effect plot

**Fig. 8:**
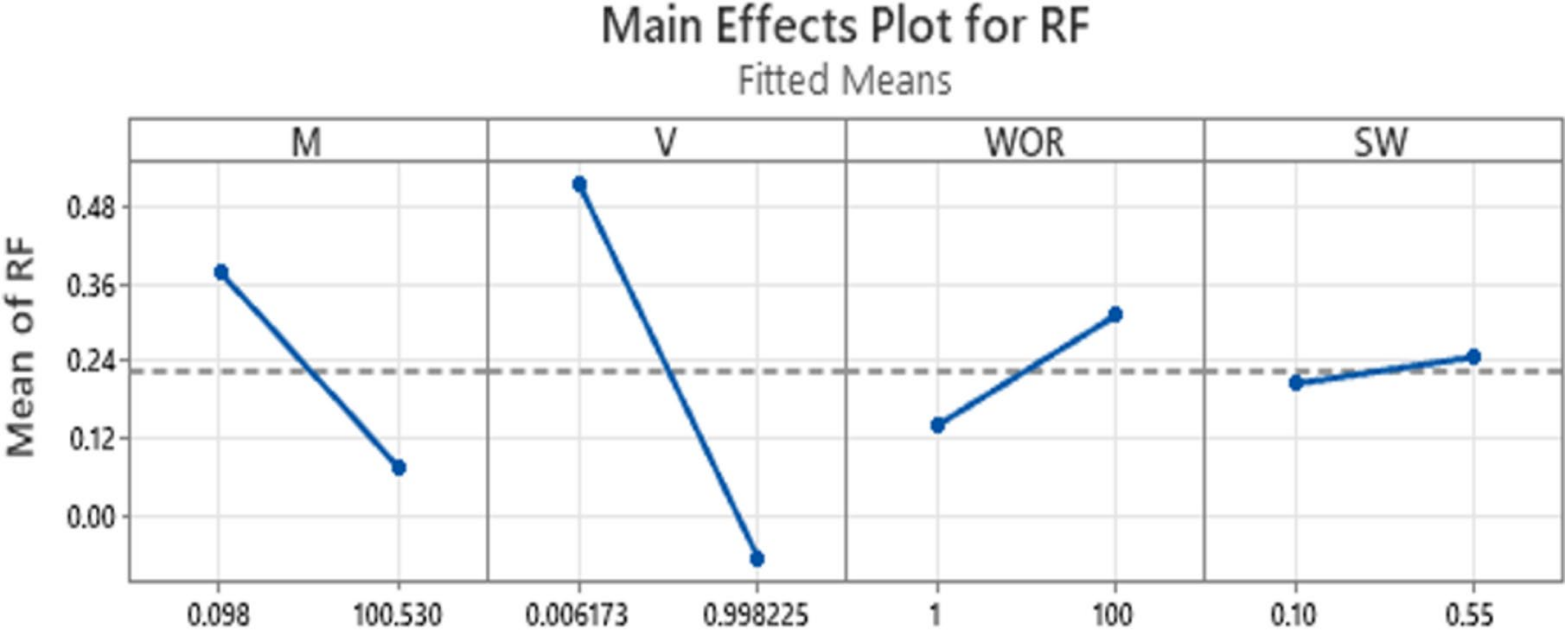
Main effect plot.

Figure  8 Presents the main effects plot for waterflooding oil recovery correlated by mobility ratio (M), reservoir permeability variation (V), water-oil production ratio (WOR), and initial water saturation (SW_i_). The horizontal line indicates the mean oil recovery value. At a low mobility ratio, oil recovery is above the mean value, but it decreases as the mobility ratio increases. Similarly, at a low permeability variation, oil recovery is above the mean value, but it declines with increasing permeability variation. Furthermore, at low water-oil production ratios, oil recovery is below the mean value; however, it improves as the water-oil production ratio increases, reaching its maximum at the highest water-oil production values. Additionally, the main effects plot suggests that initial water saturation has a relatively minor influence on oil recovery.

### Development of the machine learning models

The machine learning techniques discussed in Sect. 2.3 were applied to the dataset. Additionally, the models were evaluated and compared using various methods, such as the RMSE score and R^2^ score, to determine which model is most suitable for this dataset.

#### ANN model

The initial experiments were conducted using the artificial neural network (ANN) model. This ANN model was designed to estimate the overall oil recovery (R) based on reservoir permeability variation (V), mobility ratio (M), water-oil production ratio (WOR), and initial water saturation (SWi). In this model, there are four layers.

The first layer of the network consists of input data, featuring four neurons corresponding to reservoir permeability variation, mobility ratio, water-oil production ratio, and initial water saturation. The subsequent layers include two hidden layers, each containing 10 neurons. The final layer represents the network’s output with a single neuron for overall oil recovery. Machine learning models, especially ANN, can suffer from overfitting, where the model learns the training data too well, including noise and outliers, leading to poor generalization of new data. Overfitting often occurs due to limited datasets or overly complex model architectures. To mitigate this, techniques like cross-validation and hyperparameter tuning are used. Cross-validation involves partitioning the data into subsets and training the model multiple times to ensure consistent performance. In addition, Early stopping monitors the model’s performance on a validation set and halts training when performance degrades. To determine the optimal architecture and learning functions for the ANN model, we initially investigated the effects of varying the number of neurons (8, 9, and 10) and different transfer functions, such as tan sigmoid and logistic sigmoid, on the coefficient of determination and root-mean-square error (RMSE) in a single hidden layer. According to Table 4, the logistic sigmoid with ten neurons was chosen as the optimal transfer function due to its highest coefficient of determination (R²) of 0.9922 and the lowest RMSE of 0.0163. Subsequently, we discovered that the best accuracy (R² = 0.9994 and RMSE = 0.0047) was achieved using two hidden layers, each with 10 neurons, and the logistic sigmoid transfer function. Additionally, the pure linear function was selected for the output layer, and the Levenberg-Marquardt technique was utilized as the training algorithm. Table 4 summarizes the characteristics of the proposed model. The challenge of overfitting is notably mitigated, particularly when employing the optimized parameters for machine learning models, as detailed in Table 5. This suggests that fine-tuning the model’s parameters can effectively mitigate over-fitting, ensuring better generalization to new data.

**Table 4:** Overall oil recovery model optimization.

No. of hidden layers	Tan sigmoid function			Logistic sigmoid function			Logistic sigmoid function
	One			One			Two
No. of neurons	8	9	10	8	9	10	10/10
R^2^	0.979	0.9817	0.9901	0.9905	0.9825	0.9922	0.9994
SD	51.6522	50.2697	43.1407	35.1193	57.4868	40.8015	8.5593
RMSE	0.0267	0.0248	0.0183	0.018	0.0242	0.0163	0.0047
RE	5.6306	1.1112	3.6046	1.4276	4.6315	2.3441	-0.33
AE	21.9454	19.9949	16.9032	15.2333	23.0346	14.3462	3.504

**Table 5:** Characteristics of the overall oil recovery ANN model.

Parameter	Value
No. of layers	4
Neurons of the Input layer	4
No. of Hidden layers	2
Size of Hidden layers	10/10
Training algorithm	Levenberg-Marquardt
The hidden layer’s activation function	Logistic sigmoid
The output layer’s activation function	Pure linear

The ANN model for overall oil recovery can be described as follows:

For i = 1 to the number of neurons and j = 1 to the number of inputs, the inputs of the first hidden layer are estimated from the expression below:

11 $$\:{S}_{i,j}=\sum\:_{j=1}^{N}\left({w}_{i,j}{x}_{j}\right)+{b}_{i}\:\:\:\:\:\:\:\:\:\:\:\:\:\:\:\:\:\:\:\:\:\:\:\:\:\:\:\:\:\:\:\:\:\:\:\:\:\:\:\:\:\:\:\:$$

Where $\:{\text{x}}_{\text{j}}$ represents the normalized M, V, WOR, and S_Wi_, which can be expressed as:

12 $$M_{n} = 0.02002M - 1.00197$$

13 $$V_{n} = 2.016023V - 1.012445~$$

14 $$\:{WOR}_{n}=0.020202WOR-1.020202\:\:\:\:\:\:\:\:\:\:\:\:\:\:\:\:\:\:\:\:\:\:\:\:\:\:\:\:\:\:\:\:\:\:\:\:\:\:\:\:\:\:$$

15 $$\:{S}_{Win}=4.44444{S}_{wi}-1.44444\:\:\:\:\:\:\:\:\:\:\:\:\:\:\:\:\:\:\:\:\:\:\:\:\:\:\:\:\:\:\:\:\:\:\:\:\:\:\:\:\:\:$$

The Logistic sigmoid function is used to calculate the outputs of the first hidden layer, as illustrated below:

16 $$\:{H}_{i}=\frac{1}{1+\text{e}\text{x}\text{p}(-{S}_{i})}\:\:\:\:\:\:\:\:\:\:\:\:\:\:\:\:\:\:\:\:\:\:\:\:\:\:\:\:\:\:\:\:\:\:\:\:\:\:\:\:\:\:\:\:\:$$

The inputs of the second hidden layer are estimated as follows:

17 $$\:{SS}_{i,j}=\sum\:_{j=1}^{N}\left({w}_{i,j}{H}_{j}\right)+{b}_{i}\:\:\:\:\:\:\:\:\:\:\:\:\:\:\:\:\:\:\:\:\:\:\:\:\:\:\:\:\:\:\:\:\:\:\:\:\:\:\:\:\:\:\:\:$$

The Logistic sigmoid function is used to calculate the outputs of the second hidden layer, as illustrated below:

18 $$\:{HH}_{i}=\frac{1}{1+\text{e}\text{x}\text{p}(-{SS}_{i})}\:\:\:\:\:\:\:\:\:\:\:\:\:\:\:\:\:\:\:\:\:\:\:\:\:\:\:\:\:\:\:\:\:\:\:\:\:\:\:\:\:\:\:\:\:$$

The following function can be used to calculate the overall oil recovery:

19 $$\:R=0.439138\left[\:\:\sum\:_{i=1}^{n}\left({w}_{hoi}{HH}_{i}\right)\:+{b}_{ho}\right]+0.449751\:\:\:\:$$

The proposed model’s coefficients required to complete the calculations of overall oil recovery are presented in Tables 6, 7, 8.

**Table 6:** Weights and biases connecting the input layer to the first hidden layer.

Neuron #	$\:{w}_{i,j=1}$	$\:{w}_{i,j=2}$	$\:{w}_{i,j=3}$	$\:{w}_{i,j=4}$	$\:{b}_{i}$
1	-0.7635	-2.842	0.5567	-0.073	2.385
2	0.4096	-3.084	0.4751	-0.166	-3.684
3	-0.9356	-1.663	-4.24	-0.103	1.2335
4	2.0451	8.1095	-2.821	0.8039	-8.009
5	0.0019	0.641	0.5156	-0.543	2.6381
6	-0.2534	-2.605	5.161	-0.136	-1.211
7	-2.631	-1.606	1.0273	-0.054	-0.377
8	0.2593	-0.698	-12.47	0.105	-11.66
9	7.7251	-1.289	0.7559	-0.111	9.92
10	1.1182	-7.183	0.2954	0.1569	8.2029

**Table 7:** Weights and biases connecting the first hidden layer to the second hidden layer.

W_1_	W_2_	W_3_	W_4_	W_5_	W_6_	W_7_	W_8_	W_9_	W_10_	b
3.4266	-2.8763	0.63183	-2.1664	-1.7036	3.6072	5.3989	0.1399	3.2563	-1.1199	-4.887
-0.3042	1.5455	-1.2215	1.0358	-1.3291	-0.44493	-2.9818	1.0147	-3.9765	-3.9733	0.20448
-0.11897	-4.5513	6.0141	2.1184	-0.22865	-1.1772	-1.7491	2.7789	-2.623	-0.4364	5.2191
2.959	1.8912	2.4786	-3.6708	-1.3125	2.0565	5.522	6.7706	-14.5092	-3.8463	3.0869
-4.2268	6.3319	-0.30718	3.5636	-1.6678	5.2372	3.7586	-7.2814	-6.3568	-4.0944	2.6439
5.6314	-2.1409	6.5956	-2.7469	-5.5293	-0.25612	4.778	-0.3454	-5.2492	-3.6194	-0.57895
2.9027	9.9582	3.1199	0.26512	-11.7888	4.8972	1.9127	4.6122	-3.482	-1.7591	-1.5662
-3.7481	-2.2798	0.86981	2.0163	4.7501	2.0394	-2.3487	0.86524	3.4199	0.64739	-4.0523
3.654	-4.8861	4.0243	1.7782	-1.1766	-4.7283	-3.912	0.53242	1.3374	-0.70098	4.6162
-2.4159	-1.8223	4.4469	-2.6365	-2.6047	5.4375	12.3661	2.4237	-15.8767	-1.5808	0.44539

**Table 8:** Weights and biases connecting the second hidden layer to the output layer.

Neuron #	$\:{w}_{hoi}$	$\:{b}_{ho}$
1	-0.343	2.3095
2	-0.693	
3	-1.65	
4	-0.776	
5	-7.071	
6	-1.094	
7	8.5998	
8	-3.306	
9	1.6415	
10	12.507	

**Fig. 9:**
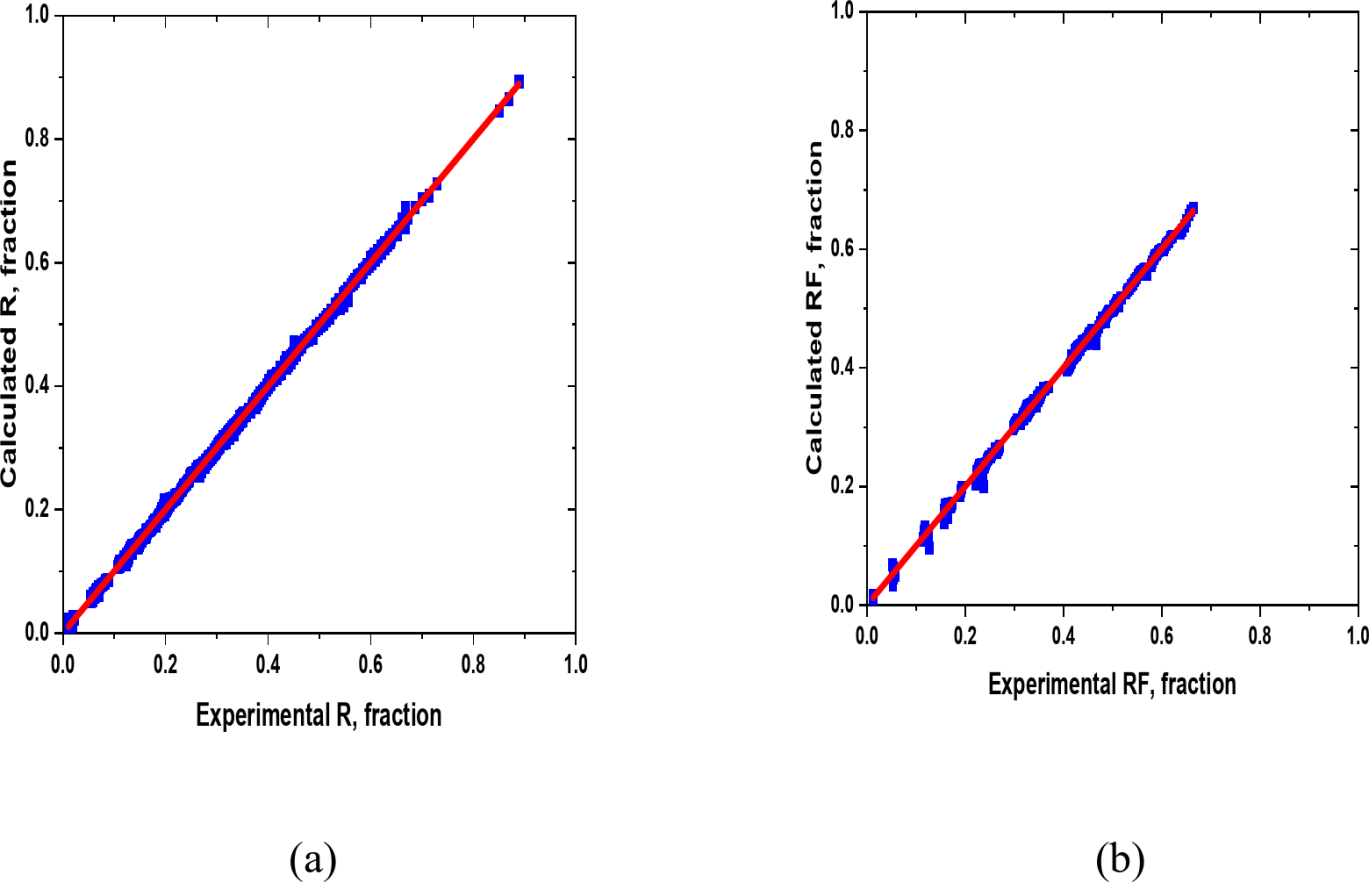

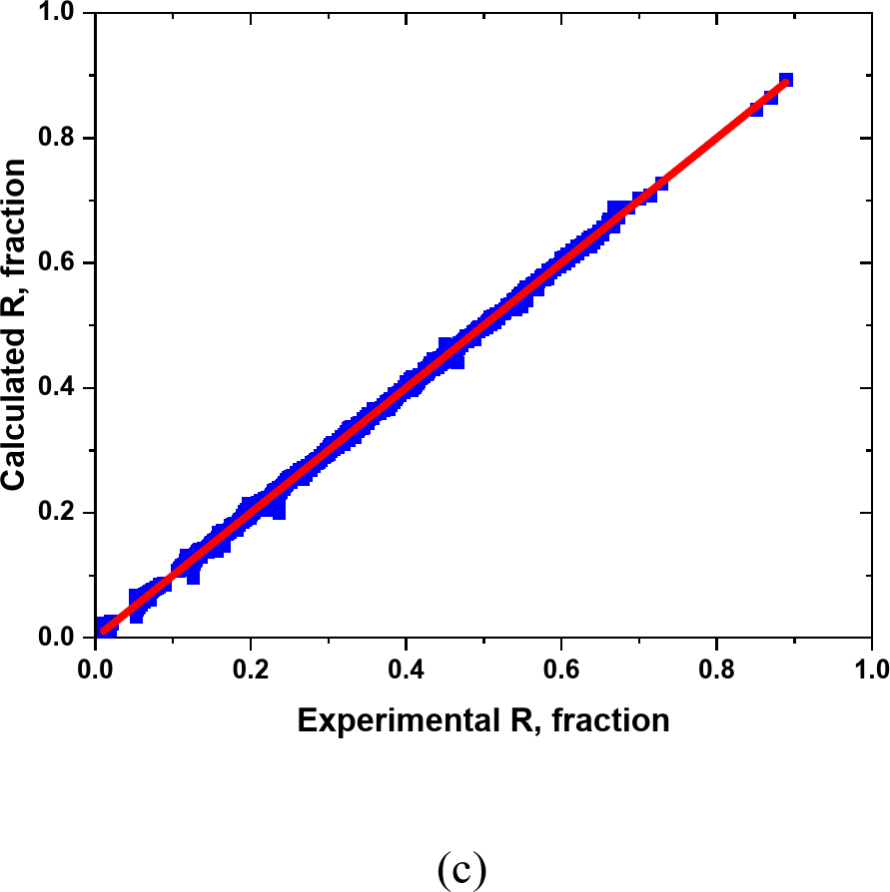
Cross plots of the overall oil recovery (R) model for (**a**) Training datasets, (**b**) Validation datasets, and (**c**) All datasets.

**Fig. 10:**
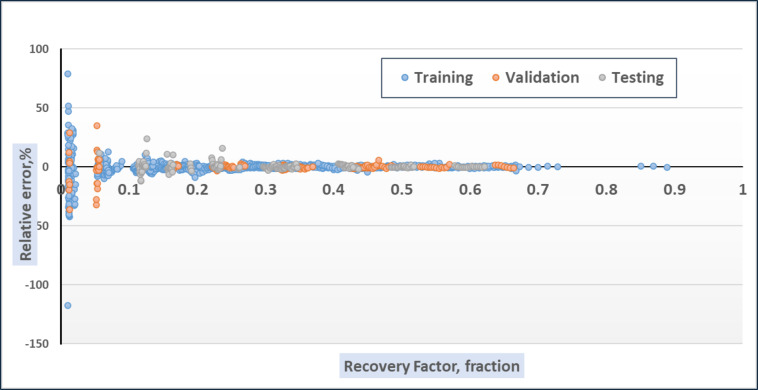
Relative error distribution for overall oil recovery (R) predicted by ANN model.

Figures 9 and 10 illustrate the regression plots for training, validation, and the entire dataset, comparing the network outputs with the target values for the overall oil recovery model. Ideally, the data points should align closely with the line of the unit slope, indicating that the network’s predictions match the target values. The results show a strong correlation between the experimental and predicted values for overall oil recovery (R), with a high R² value of approximately 0.999 for both training and validation datasets. The evaluation summary of the proposed model is tabulated in Table 9.

**Table 9:** Evaluation summary of the proposed ANN model.

Type of data	ARE	AARE	RMSE	R2
Training	-0.5451	3.9623	0.0047	0.999
Validation	-0.4434	2.9912	0.0063	0.999

#### Random forest model

A Random Forest (RF) is an ensemble of T-decision trees $\:{\left\{{h}_{t}\left(x\right)\right\}}_{t=1}^{T}$. For a given input x, each decision tree $\:{h}_{t}$ outputs a prediction. The final prediction of the RF is obtained by aggregating the individual tree predictions, often by majority voting in classification or by averaging in regression^67^.

##### Construction of each decision tree

I. Bootstrap sampling: For tree t, a bootstrap sample of size N (the number of training samples) is drawn from the original dataset $\:{\left\{\left({x}_{i},{y}_{i}\right)\right\}}_{i=1}^{N}$​. Denote this sample as $\:{D}_{t}$​.
II. Random feature subset: At each node of tree t, instead of considering all d features, a random subset $\:{F}_{t}\subset\:\left\{\text{1,2},\dots\:,d\right\}$of predefined size mmm $\:m\left(m\ll\:d\right)$ is selected to find the best split.
III. Decision tree induction: A decision tree $\:{h}_{t\:}$is grown by recursively splitting the training data (from the bootstrap sample $\:{D}_{t}$ according to the feature and split point that best separates the data in terms of the impurity measure (e.g., Gini index, entropy, etc.) in classification tasks or variance reduction in regression tasks.

##### Prediction

For a regression problem, each tree in the Random Forest outputs a real-valued prediction. The overall Random Forest prediction is the average of these individual predictions, mathematically represented as:

20 $$\:{H}_{RF}\left(x\right)=\frac{1}{T}\sum\:_{t=1}^{T}{h}_{t}\left(x\right)$$

where T is the total number of trees, and $\:{h}_{t}\left(x\right)$is the prediction from the T-th tree.

**Fig. 11:**
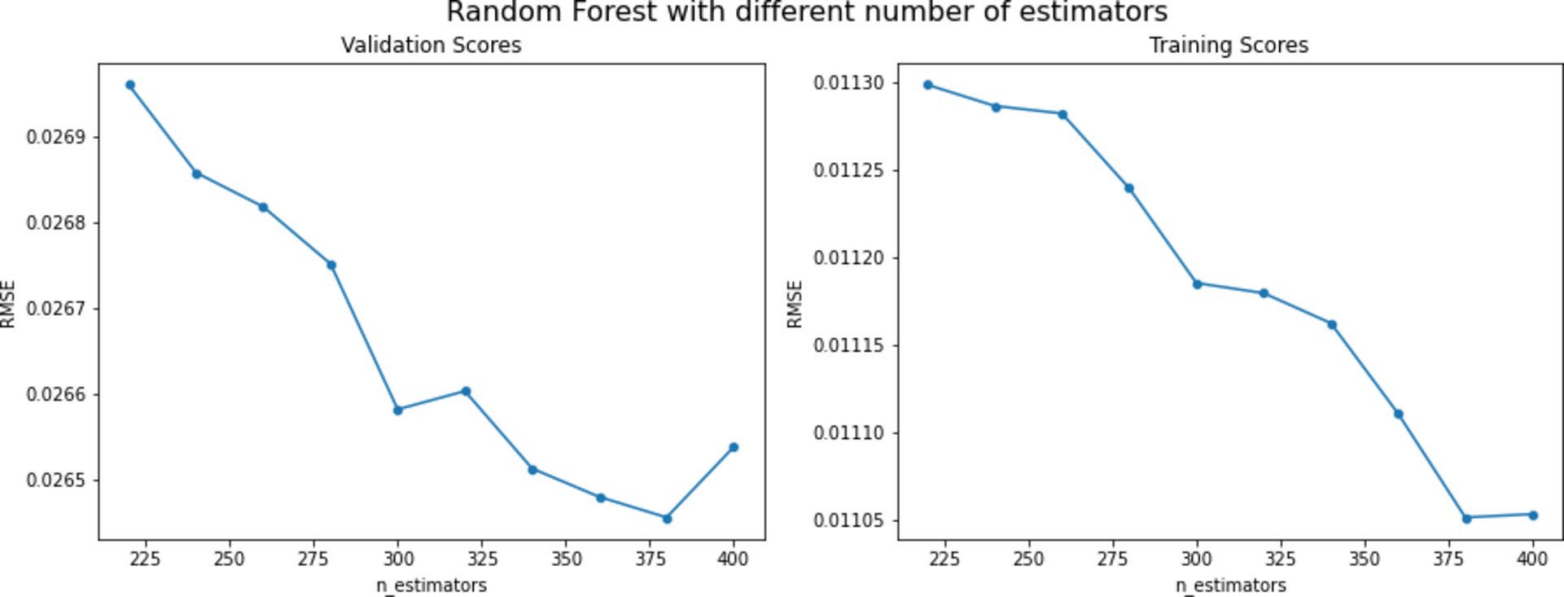
Random Forest RMSE score across different parameter values.

**Fig. 12:**
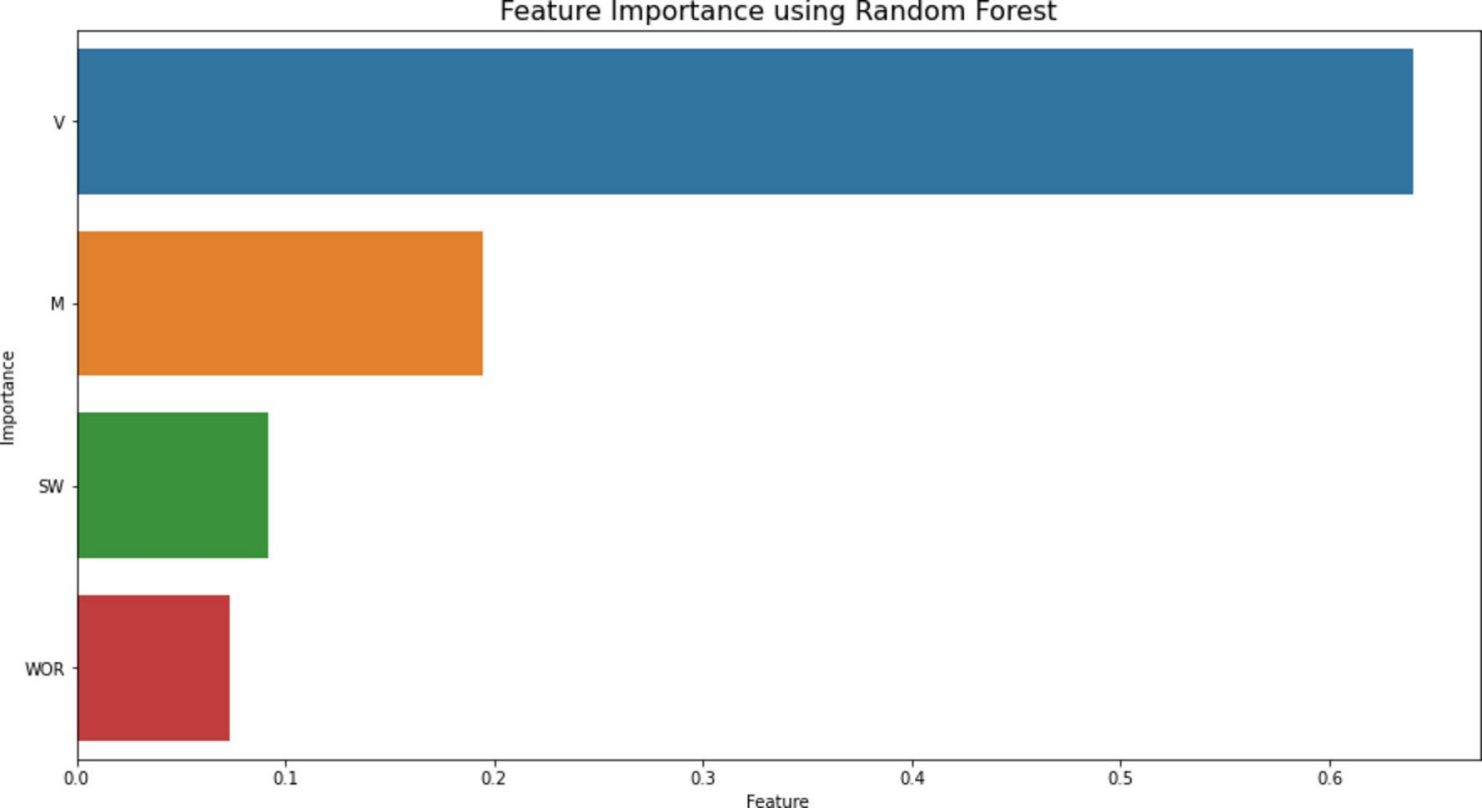
Importance of input parameters as estimated by the random forest model.

The second series of experiments utilized the random forest model. These experiments aimed to identify the optimal parameters for the model on the given dataset. The primary parameter adjusted was the number of estimators (n_estimators). Initially, this parameter was set to 220, and ten experiments were conducted with n_estimators values ranging from 220 to 400. After the experiments were finished, the RMSE score for each experiment was plotted as shown in Fig. 11; Table 10 to see which value scored the best RMSE, and Fig. 11 showed that the best n_estimaors’ value was 380, which scored an RMSE score of 0.0265. One of the main characteristics of the random forest model is that it learns how impactful and important the given features are with respect to the goal value. This is a very useful function that can be used to see which feature is the most important in the dataset. This function was used to see which feature is the most impactful and the function showed a result that feature V is the most important feature in the dataset. The feature importance was determined using the permutation importance method. It involves randomly shuffling the values of each feature and measuring the impact on the model’s performance. In addition, this method is particularly useful because it is model agnostic, meaning it can be applied to any machine learning model, including linear models, decision trees, and neural networks. The feature importance allows for a comprehensive comparison of feature importance across different models, providing deeper insights into which features are most influential in predicting the target variable. Figure 12 shows the plot of the importance of input parameters retrieved from the random forest technique. From this plot, we can infer that V and M are the most critical features for estimating the overall recovery factor of water flooding. Water saturation also contributes, but water oil ratio has the least impact on the overall recovery factor of water flooding.

**Table 10:** RMSE score for random forest across different parameter values.

n_estimators	RMSE	
	Validation	Training
220	0.02696	0.011129
240	0.02686	0.011126
260	0.02682	0.011124
280	0.02675	0.011111
300	0.02659	0.011094
320	0.02661	0.011092
340	0.02652	0.011086
360	0.02648	0.011070
380	0.02645	0.011050
400	0.02654	0.011051

#### K-nearest neighbor model

K-Nearest Neighbors (K-NN) is a non-parametric algorithm used for classification and regression. It predicts the label of a query point x based on the labels of its K closest neighbors in the training set^67^.

##### Distance metric

A common choice for the distance metric in K-NN is the Euclidean distance, which is defined as:

21 $$d\left( {x,x_{i} } \right) = \left\| {x - x_{{i2}} } \right\| = ~\sqrt {\mathop \sum \limits_{{j = 1}}^{d} \left( {z_{j} - z_{{ij}} } \right)^{2} } ~$$

where x and xi are points in a d-dimensional space, and zj and zij are the j-th components of x and xi, respectively.

##### Neighborhood definition

Given a query point x, define its neighborhood $\:{N}_{K}$(x) as the set of K training points closest to x under the chosen distance metric:

22 $$\:{N}_{k}\left(x\right)={arg}_{S\subset\:\left\{1,\dots\:N\right\},\:\left|S\right|=K},\:\sum\:_{i\in\:S}d(x,\:{x}_{i})\:\:\:\:\:\:\:\:\:\:\:\:\:\:\:\:\:\:\:\:\:\:\:\:\:\:\:\:\:\:\:\:\:\:\:\:\:\:\:\:\:\:\:\:\:\:\:\:\:\:\:\:\:\:\:\:\:\:\:\:\:\:\:\:\:\:\:\:\:\:\:\:\:\:\:$$

Where N is the total number of training points; K is the number of nearest neighbors; $\:d(x,\:{x}_{i})$ is the distance between the query point x and a training point $\:{x}_{i}$.

##### Prediction

In regression, each neighbor has a real value $\:{y}_{i}\in\:R$, and the prediction is typically the average of these values. The prediction function for K-Nearest Neighbors (KNN) regression is given by:

23 $$\:{H}_{KNN}\left(x\right)=\frac{1}{K}\sum\:_{i\in\:{N}_{K}\left(x\right)}{y}_{i}$$

where $\:{N}_{K}\left(x\right)$ represents the set of the K*K* nearest neighbors of *x.*

**Fig. 13:**
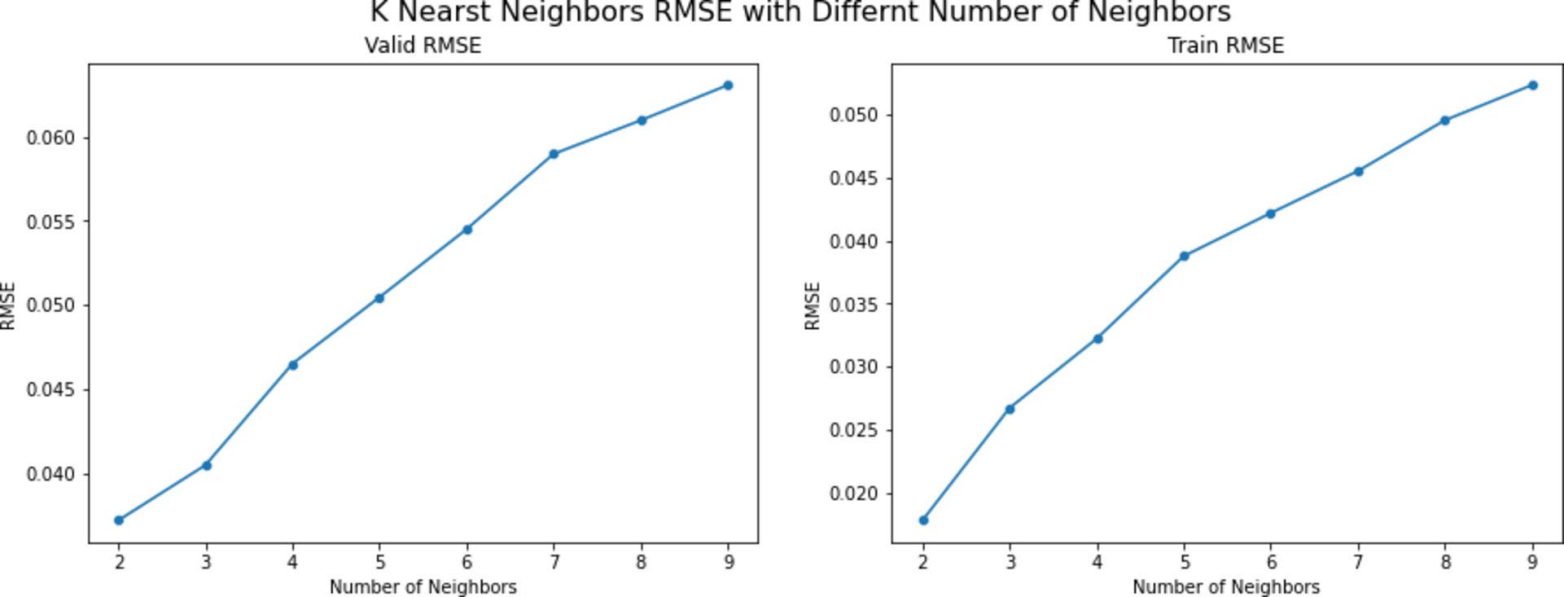
K-NN RMSE scores across different parameter values.

The third series of experiments utilized the K-Nearest Neighbor model, focusing on varying the number of neighbors (n_neighbors). This parameter determines how many neighbors are considered when comparing new data. The experiments began with n_neighbors set to 2, incrementing by 1 in each subsequent experiment until reaching 9. After completing the experiments, a figure was created to visualize the differences in RMSE scores as the n_neighbors parameter changed. The figure showed that the best value for the n_neighbors parameter is 2 due to the result given by that model, which was a 0.0372 RMSE score. Figure 13 shows the RMSE difference between each model in this set of experiments.

#### SVM model

Support Vector Machines (SVMs) aim to find a decision boundary that maximizes the margin between classes. For simplicity, the standard linear SVM for a binary classification $\:({y}_{i}\in\:\{-1,\:+1\left\}\right)$ is presented below.

##### Primal formulation

Given a training set $\:{\:\left\{\right(x\_i,y\_i\:\left)\right\}}_{i=1}^{N}$where xi ∊ Rd and yi ∊ {-1, + 1}, the soft margin SVM optimization problem in its primal form is:

24 $${}_{{w,b,\xi }}^{{\min }} ~~\frac{1}{2}\left\| {w^{2} } \right\| + C\mathop \sum \limits_{{i = 1}}^{N} \xi _{i}$$

25 $$\:subject\:to\:{y}_{i}\left({w}^{t}{x}_{i}+b\right)\ge\:1-{\xi\:}_{i},\:{\xi\:}_{i}\ge\:0,\:i=1,\dots\:.,N$$

Where: w ∊ Rd is the normal vector to the hyperplane; b ∊ R is the bias term;$\:{\xi\:}_{i}\ge\:0\:$are the slack variables allowing for soft margin;$\:and\:C>0$ is a regularization parameter that trades off margin size and classification error.

##### Decision function

Once w and b are learned, the decision function for a test point x is:

26 $$\:f\left(x\right)={w}^{t}x+b$$

*Where w* is the weight vector; *b* is the bias term; *x* is the input feature vector; $\:{w}^{t}x\:$represents the dot product of w*w* and x*x*.

The predicted label is given by:

27 $$\:{H}_{SVM}\left(x\right)=sign\left(f\left(x\right)\right)$$

##### Kernel extension

In many applications, a kernel function $\:K(x,\:{x}^{{\prime\:}})$ is introduced to handle nonlinear decision boundaries. The corresponding dual formulation leverages $\:K\left({x}_{i},\:{x}_{j}\right)$ to work in a high-dimensional (possibly infinite-dimensional) feature space without explicitly computing coordinates in that space. The prediction function in the kernelized case can be written as:

28 $$\:f\left(x\right)=\:\sum\:_{i=1}^{N}{\alpha\:}_{i}{y}_{i}\:K\left({x}_{i},\:x\right)+b\:\:\:\:\:\:\:\:\:\:\:\:\:\:\:\:\:\:\:\:\:\:\:\:\:\:\:\:\:\:\:\:\:\:\:\:\:\:\:\:\:\:\:\:\:\:\:\:\:\:\:\:\:\:\:\:\:\:\:\:\:\:\:\:\:\:\:\:\:\:\:\:\:\:\:\:$$

Where $\:{\alpha\:}_{i}$ are Lagrange multipliers obtained by solving the dual optimization problem, and only a subset of the $\:{\alpha\:}_{i}$​ (the support vectors) are non-zero.

**Fig. 14:**
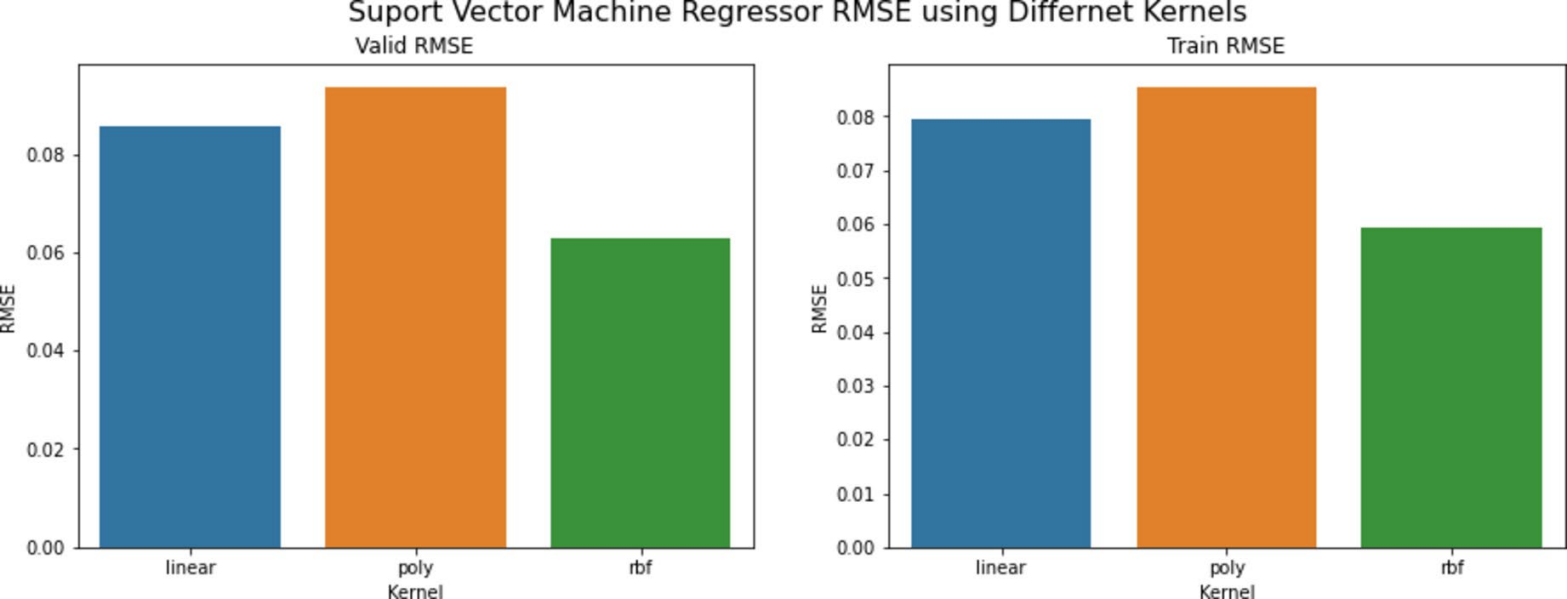
SVM RMSE scores across different kernel parameter values.

The final series of experiments aimed to identify the optimal SVM model. The main parameter that was changed in this experiment was the kernel parameter. The kernel parameter in the SVM model can take multiple values like the linear kernel, polynomial kernel, and RBF kernel. The experiments started with setting the kernel parameter to the linear kernel and starting the training. Then the polynomial and RBF kernels were set as the parameter values for the next two experiments. The results were plotted to see which kernel parameter value gave the best performance, and the result showed that the RBF kernel achieved the best performance of 0.0629 RMSE scores. Figure 14 shows the performance results based on the kernel parameter values.

### Comparison between the applied machine learning models’ performances

**Fig. 15:**
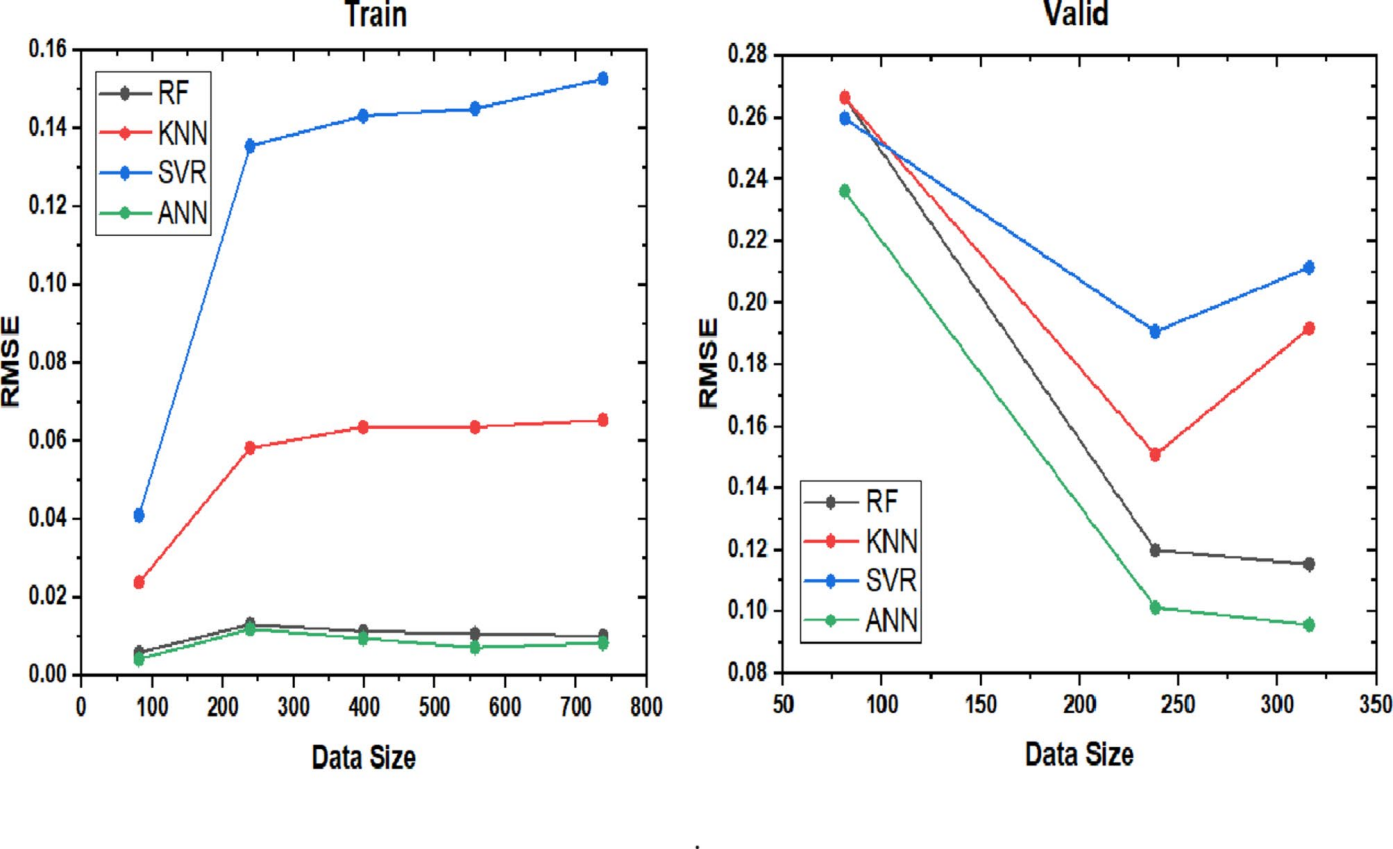
Learning curves of the best models.

Table 11 shows the tuned hyperparameters of Random Forest, K-nearest neighbors, and SVM in terms of the computational process of the developed model^68^. After obtaining the best parameter set from each model, the four techniques were compared to see which model was the best one overall. The learning curves for the four models were plotted to see the difference between the models in the learning process and which model scored the best RMSE score. The learning curves plot is a plot to show the learning behavior of the model during the training phase and the validation phase. It works by splitting the data into eight folds and testing out the performance of the model at each fold adding up to have the model performance on the whole data points at the end. Figure 15 demonstrates a comparison between the learning curves of all the implemented models. The left side of the figure shows the models’ performance during the training phase, while the right side illustrates their performance during the validation phase.

**Table 11:** Optimized hyperparameters for machine learning models used in this study.

Techniques	Hyperparameters	Range	Optimized values
Random forest (RF)	Number of trees	220–400	400
	Maximum depth	Default	None
	Samples split minimum	Default	2
	Samples leaf minimum	Default	1
	Number of Features	Default	1.0
K-nearest neighbors (KNN)	Number of Neighbors	2–9	2
	Distance metric	Default	“minkowski”
SVM	Regularization parameter; C	Default	1.0
	Kernel	“linear”, “poly”, “rbf”, “sigmoid”	“rbf”
	Gamma	Default	“scale”

The learning curve plot shows that the behaviour of the SVM is the worst among the models in both phases while the ANN achieved one of the best behaviours in both phases along with the random forest. Furthermore, the R^2^ score was used as another evaluation metric to distinguish which model is the best. The comparison showed that the ANN model has a better performance in all the metrics, with a training RMSE score of 0.0047, a validation RMSE score of 0.0063, and an R^2^ score of 0.99. The results and comparison between the models’ performances and scores are shown in Table 12. The Comparison of the developed ANN correlation with existing literature models is stated in Table 13. It looks like our model has achieved a high R² value, indicating a strong correlation between the inputs and the overall recovery factor. The RMSE values are also quite low, suggesting good model accuracy. In the present study, the ANN model outperforms the other machine learning-based models (RF, K-NN, and SVM) in terms of the coefficient of determination (R²) and root-mean-square error (RMSE) due to its ability to capture complex, non-linear relationships within the data. The ANN’s architecture, which includes multiple layers and neurons, allows it to learn and model intricate patterns and interactions among the input variables (mobility ratio, reservoir permeability variation, water-oil production ratio, and initial water saturation). This results in higher prediction accuracy and lower error rates compared to the other models. Additionally, the ANN model’s flexibility in adjusting weights and biases during training enables it to achieve better generalization and robustness in predicting oil recovery under varying reservoir conditions.

**Table 12:** Comparison between the applied machine learning models’ performances.

Applied model	Training RMSE	Validation RMSE	Validation *R* ^2^
Artificial neural network	0.004723	0.006340	0.998751
Random forest	0.010825	0.028160	0.973398
k-Nearest neighbor	0.026729	0.040473	0.947415
Support vector machine	0.059483	0.062933	0.802895

**Table 13:** Comparison of the developed ANN correlation with existing literature models.

Correlation	ANN model		Model evaluation		
	Inputs	output	RMSE	MAPE (%)	*R* ^2^
Gomaa et al.2022 ^43^	Permeability variation, Mobility ratio, and Water-oil ratio	vertical sweep efficiency	0.002		0.99
Kalam et al.2021 ^42^	Permeability variation, Mobility ratio, Anisotropy ratio, Wettability indicator, and Production of water cut	Movable recovery factor	0.0003	7.08	0.997
This work	mobility ratio, reservoir permeability variation, water-oil production ratio, and initial water saturation	Overall recovery factor	0.0063	2.9	0.99

### Analysis from a reservoir engineering standpoint

From the Feature plot, we can infer that permeability variations and mobility ratio are the most critical features for estimating the overall recovery factor of water flooding. Water saturation also contributes, but the water-oil ratio has the least impact on the overall recovery factor of water flooding. Mobility ratio (M) is defined as the ratio of the mobility of the displacing fluid (water) to the mobility of the displaced fluid (oil). When the mobility ratio is less than or equal to 1, the displacement is more stable, leading to a more efficient sweep of the oil. This condition promotes piston-like displacement, where water pushes oil uniformly, minimizing bypassing and fingering. When the mobility ratio is greater than 1, water moves faster than oil, causing instability in the displacement front. This results in water fingering and channeling through the oil, leading to early water breakthrough and reduced sweep efficiency.

Reservoir Permeability heterogeneity refers to the variations in permeability within the reservoir. These variations can significantly affect the flow of fluids during water flooding. In highly heterogeneous reservoirs, the presence of high-permeability streaks or layers can lead to uneven water distribution. Water tends to preferentially flow through high-permeability zones, bypassing oil in low-permeability zones, which reduces overall recovery efficiency. In contrast, in more homogeneous reservoirs, water flooding tends to be more uniform, leading to better sweep efficiency and higher oil recovery. Overall, Understanding and managing the oil-water mobility ratio and reservoir heterogeneity is crucial for optimizing water flooding performance and designing more effective water flooding strategies to maximize oil recovery.

The performance of water flooding in oil reservoirs is significantly influenced by water saturation and the water-oil ratio (WOR). If the reservoir has a high initial water saturation, the effectiveness of water flooding can be reduced because the water already occupies a significant portion of the pore space, leaving less room for oil displacement. A lower initial water saturation generally means more oil can be displaced by the injected water, leading to higher recovery efficiency. On the other hand, the water-oil ratio (WOR) is the ratio of the volume of water produced to the volume of oil produced. It is a key indicator of the performance of a water flood. In the early stages, the WOR is typically low, indicating that the injected water is effectively displacing oil, and the production is predominantly oil. As water flooding progresses, the WOR increases. A rising WOR indicates that more water is being produced relative to oil, which can signal water breakthroughs and the onset of water channeling. A high WOR can reduce the economic efficiency of the operation, as handling and treating large volumes of produced water can be costly. Monitoring the WOR over time helps in diagnosing the efficiency of the water flood. A sudden increase in WOR can indicate issues such as coning, channeling, or breakthrough. Practically, using tools like diagnostic WOR plots and saturation logs to continuously assess and adjust the water flooding strategy.

## Conclusion

Four machine learning models based on ANN, RF, K-NN, and SVM showed their capability of accurately predicting the overall oil recovery based on 1054 datasets of mobility ratio (M), reservoir permeability variation (V), water-oil production ratio (WOR), and initial water saturation (S_Wi_). In accordance with the results obtained, the following conclusions were drawn:

- The four proposed models of ANN, RF, K-NN, and SVM achieve low values of root-mean-square error of (0.004723, 0.010825, 0.026729, and 0.059483) and (0.006340, 0.028160, 0.040473, and 0.062933) in the case of the training and validating sets, respectively.
- The ANN model outperforms the other machine learning-based models in respect of coefficient of determination (R^2^) and root-mean-square error (RMSE).
- A new correlation has been established to estimate the overall oil recovery of water flooding using ANN.
- The coefficient of determination values between actual and estimated overall oil recovery (R) from the ANN model were found to be 0.999 compared to 0.97, 0.95, and 0.80 from the RF, K-NN, and SVM models in the case of validating sets, respectively.
- Lastly, the proposed models can be applied for estimating the performance of waterflooding operations in heterogeneous and complex reservoirs with the reservoir permeability variation (V) ranging from 0.006 to 0.998, water-oil production ratio (WOR) ranging from 1 to 100, initial water saturation ranging (S_Wi_) from 0.1 to 0.55, and mobility ratio (M) ranging from 0.098 to 100.

Overall, these ML models can provide accurate and efficient predictions by leveraging input parameters such as reservoir permeability variation, mobility ratio, water-oil production ratio, and initial water saturation. The insights gained from this study could enhance reservoir management and optimize waterflooding strategies, leading to improved oil recovery.

## Data Availability

The datasets used and/or analysed during the current study are available from the corresponding author on reasonable request.

## Abbreviations

ANN: Artificial neural network
ARE: Absolute relative error
AARE: Absolute average relative error
RF: Random forest
K-NN: K-nearest neighbor
SVM: Support vector machine
R: Overall oil recovery
M: Mobility ratio
S_Wi_: Initial water saturation
WOR: Water-oil production ratio
V: Reservoir permeability variation
R^2^: Coefficient of determination
RMSE: Root-mean-square error
SD: Standard deviation
RE: Relative error
W_i,j_: Weight of neurons i and inputs j
w_hoi_: Weight for hidden and output layer
b_i_: Bias of neuron i
b_ho_: Bias for hidden and output layer
